# IL-17 ligand-targeting inhibitors in psoriatic arthritis: a focused systematic review and network meta-analysis of efficacy, safety, and certainty of evidence

**DOI:** 10.3389/fimmu.2026.1893092

**Published:** 2026-08-13

**Authors:** Yahya Kayed AbuJwaid, Anas K. Assi, Alhareth M. Amro, Habeeb H. Awwad, Mohammed Ehmidat, Salahaldeen Deeb, Mohammad W. Awlad Mohammad, Abdallah Dwayat, Amro Odeh, Yousef Albow, Mohammad Yasini, Mohammad Alfrookh, Laith Alamlih

**Affiliations:** 1 Faculty of Medicine, Al-Quds University, Jerusalem, Palestine; 2 Faculty of Medicine, Alexandria University, Alexandria, Egypt; 3 Rheumatology department, Al-Mezan Hospital, Hebron, Palestine

**Keywords:** bimekizumab, CINeMA, IL-17 inhibitors, ixekizumab, network meta-analysis, psoriatic arthritis, randomized controlled trials, secukinumab

## Abstract

**Background:**

IL-17 pathway inhibitors are established treatments for active psoriatic arthritis (PsA), but comparative evidence within this therapeutic class remains limited. We compared the efficacy, safety, treatment rankings, and certainty of evidence for established and emerging IL-17 pathway inhibitors in adults with active PsA.

**Methods:**

We conducted a PRISMA-compliant systematic review and network meta-analysis. MEDLINE via PubMed, Embase via Ovid, CENTRAL, ClinicalTrials.gov, and the EU Clinical Trials Register were searched from January 1, 2010, to January 18, 2026. Eligible studies were phase 2 or phase 3 randomized controlled trials in adults with PsA. The primary outcome was ACR50 at Weeks 12–16. Secondary outcomes included ACR20, ACR70, PASI90, minimal disease activity, HAQ-DI, safety outcomes, Week-24 outcomes, and descriptive Week-52 outcomes. Frequentist random-effects network meta-analysis was performed; certainty was assessed using CINeMA.

**Results:**

Sixteen RCTs were included. The primary ACR50 network comprised eight RCTs and ten treatment nodes. All active treatments were superior to placebo for ACR50 at Weeks 12–16, with negligible heterogeneity (τ² = 0; I² = 0%) and no important incoherence. Ixekizumab Q2W had the highest ACR50 P-score, followed by bimekizumab 160 mg with loading and ixekizumab Q4W; however, active-treatment confidence intervals overlapped. Secondary outcomes showed consistent improvements in joint, skin, multidomain, and functional endpoints. Sonelokimab and izokibep showed favorable exploratory estimates but very low certainty. Serious adverse events and discontinuations were not clearly increased versus placebo. Bimekizumab showed a modest increase in infection risk and a higher Candida signal; IBD events were rare.

**Conclusion:**

IL-17 pathway inhibitors are effective short-term treatments for active PsA. Rankings suggest potential within-class differences, but indirect comparisons and overlapping confidence intervals limit definitive superiority claims. Candida risk and patient-specific factors should inform individualized treatment selection. The protocol was registered in PROSPERO (CRD420251156756).

**Systematic Review Registration:**

https://www.crd.york.ac.uk/PROSPERO/view/CRD420251156756, identifier CRD420251156756.

## 1 Introduction

Psoriatic arthritis (PsA) is a chronic immune-mediated inflammatory disease at the interface of rheumatology and dermatology, characterized by heterogeneous involvement of peripheral joints, axial skeleton, entheses, digits, skin, and nails (1). A recent worldwide systematic review estimated a pooled prevalence of 112 per 100,000 adults, underscoring its public-health burden (2). Because PsA commonly affects musculoskeletal, cutaneous, and functional domains simultaneously, treatment evaluation should extend beyond inflammatory suppression to include joint response, skin disease control, physical function, and multidomain disease activity (3, 4).

Contemporary PsA management has shifted toward early, targeted immunomodulation guided by disease phenotype, prognostic factors, comorbidities, previous therapy, and patient preference. Updated GRAPPA and EULAR recommendations support a domain-based approach and recognize biologic disease-modifying antirheumatic drugs, including tumor necrosis factor, IL-17, IL-23, and IL-12/23 pathway inhibitors, as key options for active disease requiring targeted therapy (5, 6). Nevertheless, treatment selection remains complex because individual PsA domains differ in therapeutic responsiveness, direct comparative evidence within the IL-17 pathway remains limited, and class-relevant safety signals may influence treatment choice.

The IL-23/IL-17 axis is central to psoriatic disease. IL-17A and IL-17F amplify inflammation across skin, synovial, entheseal, and bone-related tissues, supporting the therapeutic rationale for IL-17 pathway inhibition (7, 8). Selective IL-17A inhibitors such as secukinumab and ixekizumab have established the clinical validity of this pathway, while dual IL-17A/F blockade with bimekizumab provides a mechanistically distinct strategy because IL-17F shares overlapping pro-inflammatory activity with IL-17A (9). At the same time, IL-17 signaling is important for mucosal antifungal defence and may influence intestinal immune homeostasis, making Candida infection and inflammatory bowel disease key safety outcomes when evaluating this therapeutic class (10, 11).

Previous systematic reviews and network meta-analyses have established the efficacy of biologic and targeted therapies, including IL-17 pathway inhibitors, in active PsA (12–14). However, most previous analyses were designed primarily to compare broad therapeutic classes and therefore provided limited resolution for decision-making among individual IL-17 agents, clinically relevant dose regimens, and loading strategies. Furthermore, earlier syntheses included fewer data for dual IL-17A/F blockade and emerging IL-17-targeted agents and did not consistently integrate stringent joint response, skin response, multidomain disease control, physical function, class-relevant safety outcomes, and certainty of evidence within a single framework. The incremental contribution of the present review is therefore not to re-establish the efficacy of IL-17 inhibition as a therapeutic class, but to provide an updated, regimen-level, within-pathway comparison for situations in which IL-17 inhibition has already been considered clinically appropriate. We prioritized ACR50 at a harmonized early assessment window and evaluated joint, skin, multidomain, functional, and safety outcomes alongside treatment rankings, sensitivity analyses, subgroup findings, and CINeMA/GRADE certainty. This focused approach was intended to support more individualized selection among established and emerging IL-17 pathway treatments in adults with active PsA. Accordingly, this study aimed to compare ligand-targeting IL-17 inhibitors in adults with active PsA, using ACR50 at Weeks 12–16 as the primary endpoint and evaluating additional joint, skin, functional, minimal disease activity, safety, treatment-ranking, sensitivity, and certainty-of-evidence outcomes.

## 2 Methods

### 2.1 Protocol and registration

This systematic review was conducted and reported in accordance with the Preferred Reporting Items for Systematic Reviews and Meta-Analyses (PRISMA) 2020 guidelines (**Supplementary Table 1**) (15). The protocol was registered in PROSPERO (CRD420251156756) before formal data extraction and quantitative synthesis commenced. Protocol refinements were made before final quantitative synthesis and updated in PROSPERO. These included use of RoB 2.0, specification of Week 12–16 as the primary time window with priority to Week 16 data, use of frequentist NMA as the primary model with Bayesian sensitivity analysis, and restriction of sonelokimab and izokibep to exploratory analyses. The primary review question was unchanged.

### 2.2 Search strategy and information sources

A systematic literature search was performed in MEDLINE via PubMed, Embase via Ovid, and the Cochrane Central Register of Controlled Trials (CENTRAL) from January 1, 2010, to January 18, 2026. The search strategy was intentionally broader than the intervention eligibility criteria to maximize sensitivity and ensure identification of all potentially relevant IL-17 pathway trials. Search terms therefore included both ligand-targeting agents secukinumab, ixekizumab, bimekizumab, sonelokimab, and izokibep and the receptor-targeting agent brodalumab, together with their generic, developmental, and brand names. Records identified through this broad search were subsequently assessed against the prespecified intervention criteria described below. Thus, inclusion of brodalumab in the electronic search did not imply eligibility for the quantitative synthesis. No language restrictions were applied. The full electronic search strategies are provided in **Supplementary Table 2**.

Additional records were identified by searching ClinicalTrials.gov and the EU Clinical Trials Register. Reference lists of included studies and relevant systematic reviews were hand-searched. All search results were exported to EndNote reference management software (version 21.4). Duplicate records were automatically removed by the software.

### 2.3 Eligibility criteria

Eligible studies were randomized controlled trials (RCTs) of phase 2 or phase 3 that enrolled adults (≥18 years) with psoriatic arthritis (PsA) fulfilling the CASPAR criteria. Both placebo-controlled and active-controlled RCTs were eligible. Studies conducted primarily in psoriasis populations were eligible only if a clearly defined PsA subgroup was reported and PsA-specific joint outcomes with corresponding denominators were extractable.

Interventions of interest included established IL-17A inhibitors secukinumab and ixekizumab, the dual IL-17A/F inhibitor bimekizumab, and the emerging IL-17 pathway inhibitors izokibep and sonelokimab. Secukinumab, ixekizumab, and bimekizumab were included in the primary network, whereas izokibep and sonelokimab were evaluated only in exploratory analyses. All dose regimens, routes (subcutaneous or intravenous), and loading strategies were eligible, with separate nodes for each clinically relevant regimen. Adalimumab (a TNF inhibitor) was allowed as a bridging comparator to ensure network connectivity, but it was not the focus of interpretation. The intervention estimand was restricted to therapies that directly neutralise IL-17 ligands. Eligible agents therefore included selective IL-17A inhibitors, including secukinumab, ixekizumab, and izokibep, and dual IL-17A/F inhibitors, including bimekizumab and sonelokimab. Brodalumab was identified through the intentionally sensitive search strategy but was excluded from the eligible intervention set because it binds IL-17 receptor A rather than directly neutralizing an IL-17 ligand. As IL-17RA is a shared receptor component for several IL-17-family cytokines, receptor blockade produces a broader and pharmacologically distinct pattern of pathway inhibition and was considered outside the prespecified ligand-targeting treatment estimand. Brodalumab has demonstrated efficacy in randomized PsA trials but is not currently approved for PsA in major regulatory jurisdictions; this regulatory status was considered additional clinical context rather than the primary basis for exclusion. Brodalumab trial reports were therefore documented during full-text assessment but were not entered into the network meta-analysis. Comparators could be placebo or any active therapy. All treatment arms from eligible studies were included in the evidence network.

The primary efficacy outcome was the proportion of patients achieving ACR50 (≥50% improvement in American College of Rheumatology criteria) at Week 12–16, prioritizing Week 16 when available. When more than one assessment was available within this window, the time point closest to Week 16 was selected. A sensitivity analysis was restricted to trials reporting exact Week 16 data. Secondary outcomes were ACR20, ACR70, PASI90 (≥90% reduction in Psoriasis Area and Severity Index), Minimal Disease Activity (MDA), and the Health Assessment Questionnaire Disability Index (HAQ-DI) mean change from baseline, assessed at Week 12–16 and Week 24 ± 2 weeks. Exploratory outcomes included Week 52 endpoints (ACR50/20/70, PASI90, MDA, HAQ-DI), which were analyzed descriptively because of loss of randomization at later time points.

Safety outcomes were serious adverse events (SAEs), any infection (with a subgroup for serious infection), Candida infections (oral, esophageal, or cutaneous), new or worsening inflammatory bowel disease (IBD), and withdrawals due to adverse events. These were evaluated at Weeks 12–16 and Week 24 of the double-blind period.

### 2.4 Study selection

After deduplication, two reviewers (MA and AD) independently screened titles and abstracts against the eligibility criteria. Potentially relevant articles were retrieved in full and independently assessed by two reviewers (HA and AO). Disagreements at either stage were resolved by consensus or by consulting a third reviewer (YKA). The selection process was documented in a PRISMA flow diagram.

### 2.5 Data extraction

Two reviewers (AA and YA) independently extracted data using a standardized, piloted form. For each arm of every study, we recorded: trial characteristics (author, year, acronym, phase, design, NCT number, publication type), baseline demographics (number randomized, age, sex, disease duration, prior biologic exposure, concomitant methotrexate use), treatment details (drug, dose, frequency, route, loading regimen), follow-up duration, and all available outcome assessment time points up to Week 52. For binary outcomes, we extracted the number of patients achieving the endpoint and the number of patients randomized (or, where not available, the number included in the primary analysis population). For continuous outcomes (HAQ-DI), we extracted the mean change from baseline (or least-squares mean change) and its standard deviation (SD) or standard error (SE). When SE was reported, it was converted to SD by multiplying the SE by the square root of the corresponding arm-specific sample size. When continuous data were reported as medians with interquartile ranges, they were converted to means and SDs using the method proposed by Abbas et al. (16). Several trials reported HAQ-DI values using non-standard decimal separators (e.g., middle dots “·”), which were normalized prior to extraction. When additional or missing study data were required, authors or sponsors were contacted to obtain the relevant files or data. Discrepancies were resolved by a third reviewer (YKA).

### 2.6 Risk of bias assessment

The risk of bias of each included RCT was assessed independently by two reviewers using the revised Cochrane Risk of Bias tool (RoB 2.0). Domains evaluated were: (1) randomization process, (2) deviations from intended interventions, (3) missing outcome data, (4) measurement of the outcome, and (5) selection of the reported result. Each domain and the overall study were rated as “low risk of bias”, “some concerns”, or “high risk”. Assessments were performed at the outcome level for all outcomes included in the synthesis, and discrepancies were resolved by a third reviewer (YKA) (17). Results were visualized with traffic-light plots and summary bar charts using the robvis package (18).

### 2.7 Data synthesis

All quantitative analyses were performed in R. A frequentist random-effects network meta-analysis was implemented using the graph-theoretical approach (Rücker-Schwarzer method) in the netmeta package. For binary outcomes, treatments were compared with odds ratios (OR) and 95% confidence intervals (CI). For HAQ-DI, SMDs were used because studies differed in reporting formats and variance structures across trials, and SMDs allowed consistent synthesis of change-from-baseline estimates. Continuity corrections of 0.5 were applied to studies with zero events in any arm. Studies with zero events in all arms for a given outcome were retained in descriptive summaries but did not contribute information to relative effect estimation. A common between-study variance (τ²) was assumed across comparisons.

The primary analysis network included placebo, secukinumab (150 mg SC with and without loading, 300 mg SC, 3 mg/kg IV), ixekizumab (80 mg Q2W and Q4W), bimekizumab (160 mg SC with and without loading), and adalimumab (bridge node). Bimekizumab 16 mg and 320 mg, secukinumab 75 mg, and the high-dose intravenous secukinumab regimen from the proof-of-concept study were considered non-standard, non-licensed, or not clinically relevant to current PsA dosing and were excluded from all NMAs. For each outcome, a separate NMA was performed when the network was connected; otherwise, the largest connected subnetwork containing placebo was used. Treatments outside the connected network were summarized descriptively. Networks were visualized with netgraph.

Heterogeneity was quantified with τ², I², and the Q statistic. Inconsistency between direct and indirect evidence was assessed globally using the design-by-treatment interaction model (Q statistics) and locally by node-splitting (netsplit), reporting relevant disagreement estimates and p-values where applicable. Small-study effects were examined with comparison-adjusted funnel plots. Egger’s test was planned when at least 10 studies were available; when applied to smaller networks, results were considered exploratory and interpreted with caution.

Treatment rankings were expressed as frequentist P-scores, the frequentist analogue of SUCRA, computed with netrank. Higher P-scores indicate a more favorable relative treatment ranking. All rankings were presented as percentages (0–100%).

### 2.8 Sensitivity analyses

Ten sensitivity analyses were performed to evaluate the robustness of the primary outcome (ACR50, Week 12–16):

1. Exact Week 16 only, excluding studies that reported only Week 12.
2. Collapsing secukinumab 150 mg loading and no-loading arms into a single node.
3. Excluding intravenous secukinumab (INVIGORATE-2).
4. Excluding BE OPTIMAL, the largest bimekizumab trial.
5. Excluding FUTURE 5, the largest secukinumab trial.
6. Excluding SPIRIT-P1, the largest ixekizumab trial.
7. Fixed-effect model.
8. Inclusion of exploratory agents (sonelokimab and izokibep) in the network.
9. Full leave-one-out analysis, iteratively removing one study at a time.
10. Collapsing all secukinumab, ixekizumab, and bimekizumab doses into single drug-class nodes.

For sensitivity analyses that collapsed dosing nodes within the same study, event counts and sample sizes were summed before pairwise contrasts were built.

### 2.9 Subgroup analyses

Subgroup NMAs were conducted for studies that enrolled exclusively biologic-naïve patients (bDMARD-naïve) and for those that enrolled exclusively TNFi-inadequate responder (TNFi-IR) patients. Studies were classified using the baseline characteristics table. A tolerance of ±2 participants was allowed only to reconcile minor rounding discrepancies between reported subgroup counts and total randomized denominators; it did not alter prespecified subgroup classification. For the naïve subgroup, a connected NMA was feasible; for the TNFi-IR subgroup, the network was disconnected and only descriptive pairwise results are reported. Studies enrolling mixed biologic-naïve and biologic-experienced populations were retained in the overall analysis but were not included in subgroup-specific NMAs unless subgroup-specific outcome data were extractable. Effect modification could not be formally assessed via network meta-regression because fewer than ten studies were available.

### 2.10 Safety analyses

Safety outcomes were analyzed at two-time windows: the primary early period (Weeks 12–16) and the extended double-blind period (Week 24). For each outcome, a random-effects NMA was attempted using the same methods as for efficacy. Because Candida events were sparse, the key comparison of bimekizumab 160 mg SC versus placebo was additionally analyzed using a fixed-effect Peto OR. Network estimates for Candida were considered exploratory and interpreted with caution. IBD events were too rare for any meaningful comparative meta-analysis and are presented descriptively as raw counts and proportions. When denominators were not reported in the original publication, the number randomized to the corresponding arm was used.

### 2.11 Exploratory analysis

Sonelokimab (ARGO trial) and izokibep (phase 2 trial) were added to the primary ACR50 network in an exploratory NMA. Treatment rankings were not computed for these exploratory agents. Their findings were considered hypothesis-generating because of limited phase 2 evidence, small sample sizes, and exploratory network placement; certainty was rated as very low in the certainty-of-evidence summary.

### 2.12 Benefit–risk composite

A pre-specified exploratory benefit–risk analysis was conducted at Week 16. P-scores from the primary NMA for ACR50, PASI90, and MDA (benefit domains), and inverted P-scores from the safety NMAs for SAE and Candida (risk domains, where higher inverted scores indicate lower risk), were combined into a weighted composite score ranging from 0 (worst) to 100 (best). Three weighting scenarios were applied: (1) Balanced: ACR50 = 0.40, PASI90 = 0.15, MDA = 0.15, SAE = 0.20, Candida = 0.10; (2) Efficacy-first: ACR50 = 0.55, PASI90 = 0.15, MDA = 0.15, SAE = 0.10, Candida = 0.05; and (3) Safety-first: ACR50 = 0.25, PASI90 = 0.10, MDA = 0.10, SAE = 0.40, Candida = 0.15. IBD was excluded because of insufficient events. The analysis was restricted to treatments for which all five component scores were available. This composite was exploratory, was not used to define primary conclusions, and was not incorporated into CINeMA/GRADE certainty ratings. Because the composite was based on relative rankings rather than absolute treatment effects, it was interpreted only as a descriptive decision-support analysis.

### 2.13 Bayesian sensitivity analysis

As a further sensitivity check, a Bayesian random-effects NMA was fitted for the primary outcome using the gemtc package, which interfaces with JAGS (Just Another Gibbs Sampler). A consistency model with a binomial likelihood and logit link was employed. Default vague priors in gemtc were used for treatment effects and heterogeneity parameters. Four MCMC chains were run, each for 50,000 iterations after a 5,000-iteration burn-in, with thinning every 10 samples. Convergence was assessed by visual inspection of trace plots and by the Gelman-Rubin diagnostic (R-hat <1.1). Posterior medians and 95% credible intervals (CrI) were obtained for all treatment effects.

### 2.14 Certainty of evidence

The certainty of evidence was evaluated using the CINeMA (Confidence In Network Meta-Analysis) framework, which adapts GRADE principles to NMA. Six domains were considered: within-study bias (overall RoB 2.0 judgement), reporting bias (evaluated by funnel-plot symmetry and trial registration status), indirectness (appropriateness of populations, interventions, and outcomes to the research question), imprecision (sample size and confidence intervals), heterogeneity (τ² and I²), and incoherence (node-splitting p-values and the global design-by-treatment test). Each domain was rated as “not serious”, “serious”, or “very serious”, and the overall certainty was rated as high, moderate, low, or very low. Certainty was summarized for the primary and secondary efficacy analyses, safety outcomes, longer-term analyses, and exploratory agents where applicable.

### 2.15 Software

Analyses were performed using R version 4.5.3 and the packages netmeta (frequentist NMA), meta and metafor (pairwise meta-analyses), gemtc and rjags (Bayesian analysis), ggplot2 and patchwork (figures), flextable and officer (tables), robvis (risk-of-bias graphics), tidyverse for data manipulation, and DiagrammeR and rsvg for the PRISMA diagram. JAGS version 4.3.2 was used for Bayesian computations.

## 3 Results

### 3.1 Study selection and characteristics

**Figure 1:**
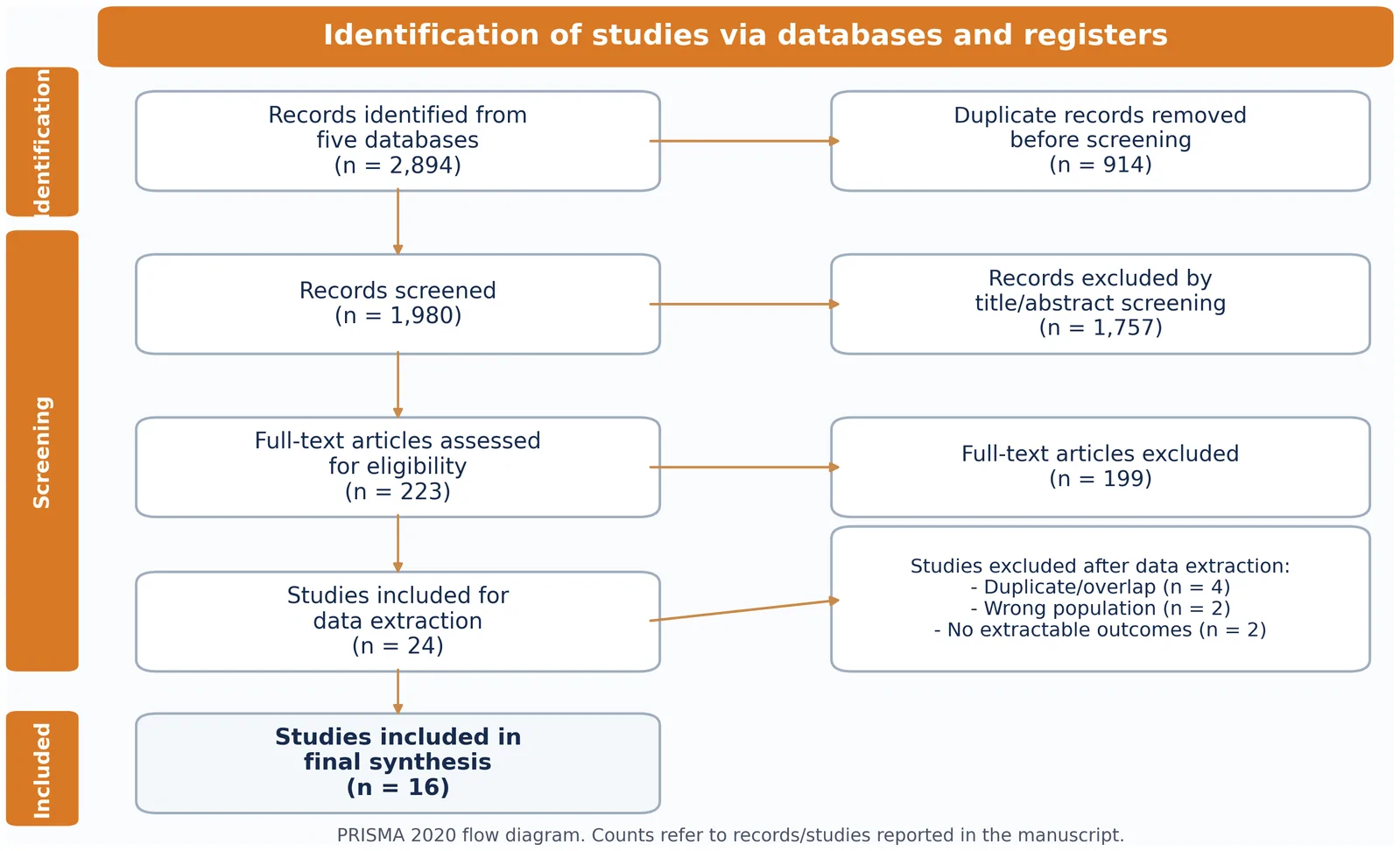
PRISMA 2020 flow diagram.

A total of 2,894 records were identified through database and registry searches. After removing 914 duplicates, 1,980 records were screened by title and abstract, and 1,757 were excluded. Of 223 full-text articles assessed for eligibility, 199 were excluded. Twenty-four studies were eligible for data extraction; eight were subsequently excluded due to duplicate or overlapping populations (n = 4), wrong population (n = 2), or no extractable outcomes (n = 2). Sixteen studies were included in the final synthesis. The complete study selection process is presented in the PRISMA flow diagram (**Figure 1**).

Ultimately, 16 full-text RCTs were included in the qualitative synthesis, and all 16 contributed data to at least one network meta-analysis (19–34). The main study and patient characteristics are presented in **Supplementary Appendix S3**. Most trials were phase 3 (n = 13), and three were phase 2/2a/2b. Eight trials evaluated secukinumab (FUTURE 1–5, MAXIMISE, INVIGORATE-2, and EXCEED), three evaluated ixekizumab (SPIRIT-P1, SPIRIT-P2, and SPIRIT-H2H), and three evaluated bimekizumab (BE ACTIVE, BE COMPLETE, and BE OPTIMAL). One phase 2 trial investigated sonelokimab (ARGO), and one investigated izokibep. Adalimumab was used as an active comparator in five RCTs (EXCEED, SPIRIT-P1, BE OPTIMAL, SPIRIT-H2H, and ARGO).

Across trials, all participants fulfilled CASPAR criteria for psoriatic arthritis. Most studies predominantly enrolled biologic-naïve patients; three trials included only tumor necrosis factor inhibitor (TNFi) inadequate responders (BE COMPLETE, SPIRIT-P2, and the 52-week dataset from FUTURE 2), and FUTURE 5 enrolled a mixed population of biologic-naïve and TNFi-experienced patients. Concomitant methotrexate use varied between treatment arms, ranging from 0% to approximately 50%.

### 3.2 Risk of bias

Risk-of-bias assessments are summarized in **Supplementary Appendix S4A, B**. Overall, 50.0% of studies were judged to be at low risk of bias, 25.0% as having some concerns, and 25.0% as high risk. Domain-level risk was lowest for measurement of the outcome, with 100% of studies judged as low risk. Risk of bias was also generally low for the randomization process and deviations from intended interventions, with 87.5% of studies judged as low risk in these domains. Greater variability was observed for missing outcome data and selection of the reported result, where high-risk judgements were identified in 6.3% and 18.8% of studies, respectively.

### 3.3 Primary efficacy – ACR50 at Week 12–16

**Figure 2:**
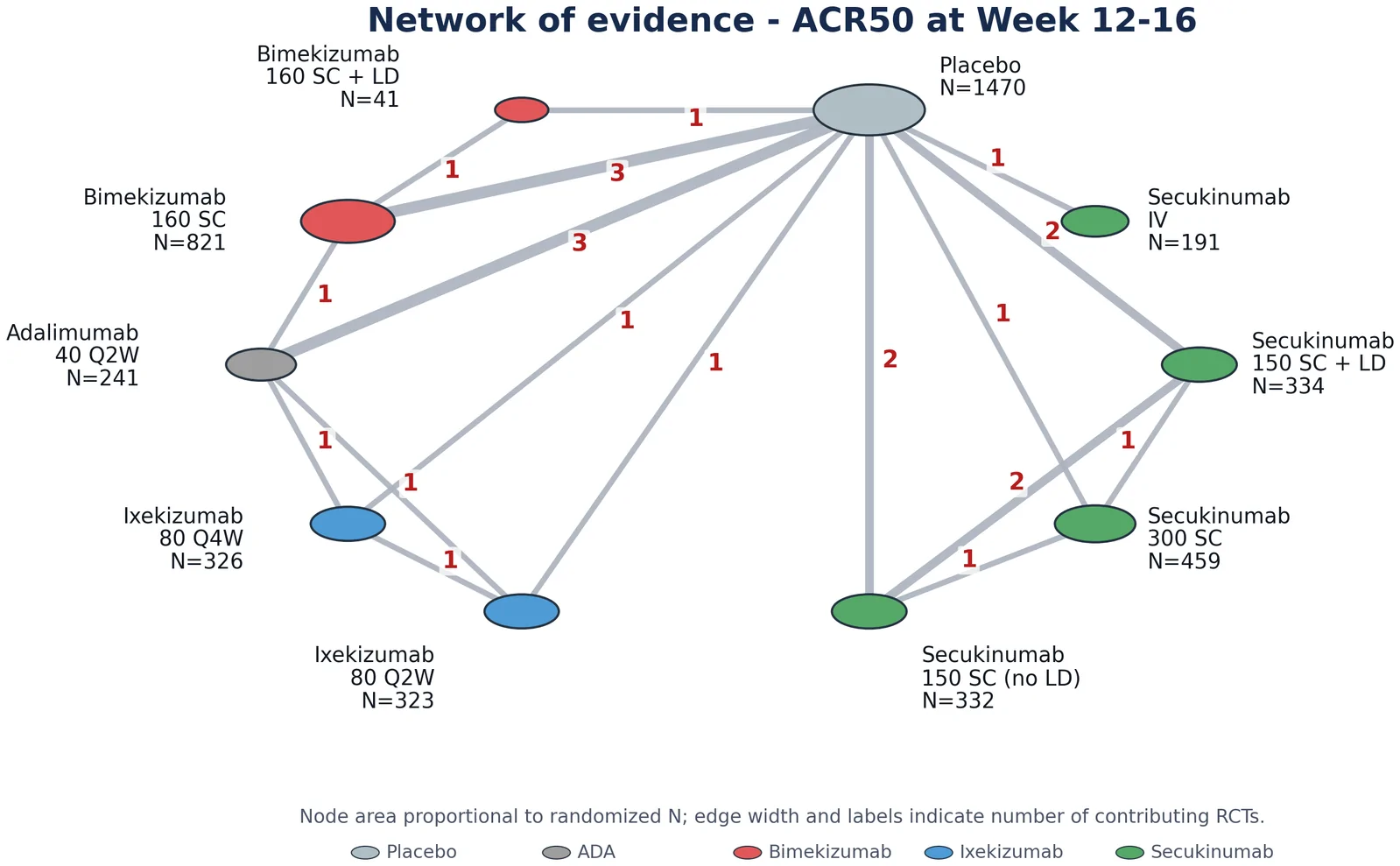
Network of evidence - ACR50 at Week 12-16.

**Figure 3:**
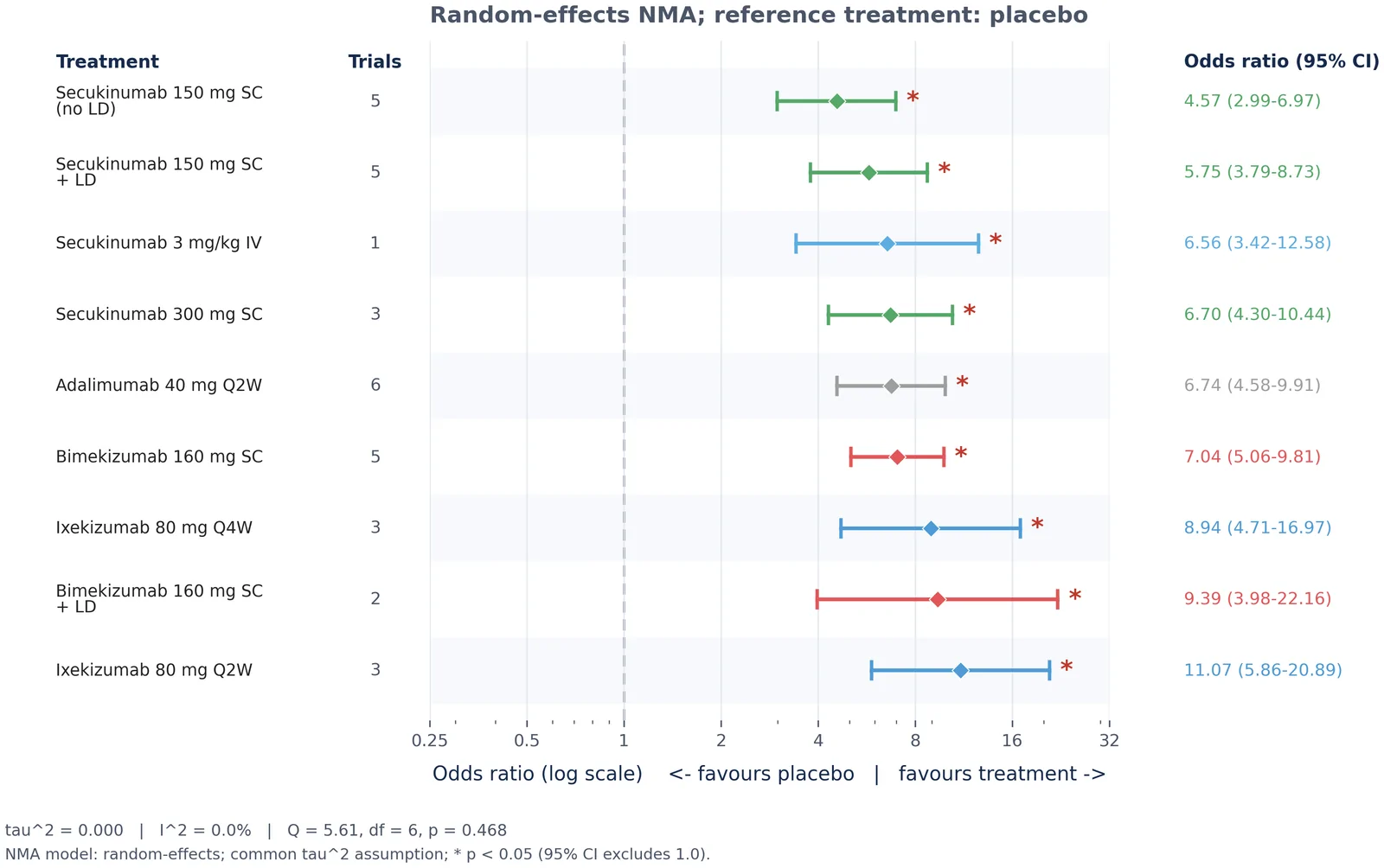
Primary NMA forest plot - ACR50 at Week 12-16.

The primary ACR50 network at Week 12–16 comprised eight RCTs and ten treatment nodes; the network geometry is depicted in **Figure 2**. Detailed effect estimates are provided in **Figure 3** and **Table 1**. The random-effects model demonstrated negligible between-study heterogeneity, with τ² = 0 and I² = 0% for the primary outcome. All active treatments were significantly superior to placebo for achieving ACR50 at Week 12–16.

**Table 1:** League table - ACR50 at Week 12-16. Random-effects network meta-analysis; odds ratios with 95% confidence intervals.

Treatment	ADA 40 Q2W	BKZ 160 SC	BKZ 160 SC + LD	IXE 80 Q2W	IXE 80 Q4W	Placebo	SEC 150 SC + LD	SEC 150 SC (no LD)	SEC 300 SC	SEC 3 mg/kg IV	P-score (%)
Adalimumab 40 Q2W	Ref	0.96 (0.67-1.36)	0.72 (0.30-1.74)	0.61 (0.35-1.07)	0.75 (0.43-1.33)	6.74 (4.58-9.91)	1.17 (0.66-2.07)	1.48 (0.83-2.62)	1.01 (0.56-1.81)	1.03 (0.48-2.19)	49.9
Bimekizumab 160 SC	1.05 (0.74-1.48)	Ref	0.75 (0.33-1.71)	0.64 (0.34-1.20)	0.79 (0.41-1.50)	7.04 (5.06-9.81)	1.22 (0.72-2.09)	1.54 (0.90-2.64)	1.05 (0.60-1.83)	1.07 (0.52-2.23)	55.8
Bimekizumab 160 SC + LD	1.39 (0.58-3.38)	1.33 (0.58-3.04)	Ref	0.85 (0.30-2.37)	1.05 (0.37-2.95)	9.39 (3.98-22.16)	1.63 (0.63-4.24)	2.06 (0.79-5.36)	1.40 (0.53-3.68)	1.43 (0.49-4.20)	74.5
Ixekizumab 80 Q2W	1.64 (0.94-2.87)	1.57 (0.83-2.97)	1.18 (0.42-3.30)	Ref	1.24 (0.70-2.19)	11.07 (5.86-20.89)	1.92 (0.90-4.11)	2.42 (1.13-5.20)	1.65 (0.76-3.58)	1.69 (0.68-4.19)	88.6
Ixekizumab 80 Q4W	1.33 (0.75-2.34)	1.27 (0.67-2.41)	0.95 (0.34-2.67)	0.81 (0.46-1.43)	Ref	8.94 (4.71-16.97)	1.55 (0.72-3.34)	1.96 (0.91-4.22)	1.33 (0.61-2.91)	1.36 (0.55-3.40)	73.8
Placebo	0.15 (0.10-0.22)	0.14 (0.10-0.20)	0.11 (0.05-0.25)	0.09 (0.05-0.17)	0.11 (0.06-0.21)	Ref	0.17 (0.11-0.26)	0.22 (0.14-0.33)	0.15 (0.10-0.23)	0.15 (0.08-0.29)	0.0
Secukinumab 150 SC + LD	0.85 (0.48-1.51)	0.82 (0.48-1.39)	0.61 (0.24-1.59)	0.52 (0.24-1.11)	0.64 (0.30-1.38)	5.75 (3.79-8.73)	Ref	1.26 (0.90-1.76)	0.86 (0.59-1.24)	0.88 (0.40-1.90)	37.1
Secukinumab 150 SC (no LD)	0.68 (0.38-1.20)	0.65 (0.38-1.11)	0.49 (0.19-1.27)	0.41 (0.19-0.88)	0.51 (0.24-1.10)	4.57 (2.99-6.97)	0.79 (0.57-1.11)	Ref	0.68 (0.47-0.99)	0.70 (0.32-1.51)	17.4
Secukinumab 300 SC	0.99 (0.55-1.79)	0.95 (0.55-1.66)	0.71 (0.27-1.88)	0.61 (0.28-1.31)	0.75 (0.34-1.63)	6.70 (4.30-10.44)	1.16 (0.81-1.68)	1.47 (1.01-2.13)	Ref	1.02 (0.46-2.24)	53.3
Secukinumab 3 mg/kg IV	0.97 (0.46-2.08)	0.93 (0.45-1.93)	0.70 (0.24-2.05)	0.59 (0.24-1.47)	0.73 (0.29-1.83)	6.56 (3.42-12.58)	1.14 (0.53-2.47)	1.44 (0.66-3.12)	0.98 (0.45-2.15)	Ref	49.7

Lower triangle = OR for row treatment vs. column treatment. Upper triangle = reciprocal OR for column treatment vs. row treatment. ADA, adalimumab; BKZ, bimekizumab; IXE, ixekizumab; SEC, secukinumab; LD, loading dose; SC, subcutaneous; IV, intravenous; Q2W/Q4W, every 2/4 weeks. Higher P-score indicates greater probability of better ACR50 ranking. Model: random-effects NMA, common tau^2 assumption.

P-score is the frequentist SUCRA analogue.

Colors distinguish treatment groups/rankings.

Compared with placebo, the odds ratios (ORs) for ACR50 were 11.07 (95% CI 5.86–20.89) for ixekizumab 80 mg every 2 weeks (Q2W), 9.39 (3.98–22.16) for bimekizumab 160 mg SC with loading, 8.94 (4.71–16.97) for ixekizumab 80 mg every 4 weeks (Q4W), and 7.04 (5.06–9.81) for bimekizumab 160 mg SC. Secukinumab 300 mg SC had an OR of 6.70 (4.30–10.44), intravenous secukinumab 3 mg/kg had an OR of 6.56 (3.42–12.58), secukinumab 150 mg with loading had an OR of 5.75 (3.79–8.73), and secukinumab 150 mg without loading had an OR of 4.57 (2.99–6.97). Adalimumab also significantly improved ACR50 responses, with an OR of 6.74 (4.58–9.91) versus placebo.

Ranking results derived from P-scores, the frequentist analogue of SUCRA, are shown in **Supplementary Figure 5**. Ixekizumab 80 mg Q2W had the highest P-score (88.6%), followed by bimekizumab 160 mg with loading (74.5%), ixekizumab Q4W (73.8%), bimekizumab 160 mg SC (55.8%), and secukinumab 300 mg SC (53.3%). Adalimumab and intravenous secukinumab had intermediate rankings (49.9% and 49.7%, respectively), whereas secukinumab 150 mg with loading (37.1%) and without loading (17.4%) ranked lower among active agents. Placebo ranked last with a P-score of 0.0%.

### 3.4 Secondary efficacy outcomes at Week 12–16

Secondary outcomes at Week 12–16 included ACR20, ACR70, PASI90, minimal disease activity (MDA), and change in Health Assessment Questionnaire Disability Index (HAQ-DI). The main network results for these endpoints are summarized in **Supplementary Figures 6A–D**.

For ACR20, the pattern of treatment effects closely mirrored that seen for ACR50. All IL-17 inhibitors and adalimumab significantly improved ACR20 responses compared with placebo. Bimekizumab 160 mg SC yielded the highest OR versus placebo at 6.66 (95% CI 3.91–11.33), followed by other active agents with ORs generally in the range of approximately 3–5. For ACR70, ORs were numerically larger than for ACR20 and ACR50 because of lower placebo response rates; bimekizumab 160 mg with loading achieved an OR of 12.02 (3.33–43.39) versus placebo, while other IL-17 inhibitors and adalimumab also showed significant improvements relative to placebo.

Skin outcomes based on PASI90 showed particularly large treatment effects. Ixekizumab Q4W and Q2W produced the largest ORs versus placebo, at 30.93 (10.32–92.73) and 25.97 (8.63–78.13), respectively. Bimekizumab 160 mg SC was also associated with a substantial PASI90 benefit (OR 15.45, 8.07–29.60), while secukinumab regimens and adalimumab showed robust, though comparatively smaller, improvements. SUCRA-based rankings for PASI90 placed ixekizumab Q4W and Q2W at the top, followed by bimekizumab 160 mg and other IL-17 inhibitors.

For MDA, the network meta-analysis indicated that bimekizumab 160 mg SC was most likely to achieve MDA (OR 5.96, 95% CI 3.87–9.18 vs placebo), with secukinumab 300 mg SC also performing strongly (OR 5.40, 2.91–10.00). Adalimumab and other IL-17 regimens produced moderate-to-large improvements in MDA probability compared with placebo. P-score rankings for MDA placed bimekizumab 160 mg at the top, with secukinumab 300 mg and intravenous secukinumab also ranking highly among active comparators.

HAQ-DI outcomes were expressed as standardized mean differences (SMDs) versus placebo. Negative SMDs favor active treatment. All IL-17 regimens and adalimumab produced small-to-moderate improvements in physical function compared with placebo. The SMDs for selected regimens were –0.63 (95% CI –0.80 to –0.45) for bimekizumab 160 mg, –0.54 (–0.93 to –0.16) for ixekizumab Q2W, –0.66 (–1.14 to –0.17) for ixekizumab Q4W, –0.67 (–1.20 to –0.14) for secukinumab 300 mg, and –0.82 (–1.46 to –0.17) for secukinumab 150 mg. In contrast with the joint response endpoints, heterogeneity for HAQ-DI was substantial (I² = 80.5%), indicating greater between-study variability in functional outcomes. For other secondary endpoints (ACR20, ACR70, PASI90, MDA), between-study heterogeneity ranged from low to moderate, with τ² values from 0 to 0.17 and I² values between 0% and 36%.

### 3.5 Network consistency

Assessment of local and global consistency did not identify evidence of incoherence in the primary ACR50 network. Node-splitting analyses did not reveal statistically significant inconsistency between direct and indirect comparisons for any treatment contrast (all p > 0.10), as summarized in **Supplementary Figure 7**. The global design-by-treatment interaction test also indicated no important inconsistency, with a Q statistic of 5.61 on 6 degrees of freedom (p = 0.468). Design-specific and between-design decomposition of Q similarly showed no evidence that any particular design, including placebo versus adalimumab or placebo versus bimekizumab, materially drove inconsistency.

### 3.6 Exploratory analysis of sonelokimab and izokibep

An exploratory network meta-analysis including the phase 2 trials of sonelokimab (ARGO) and izokibep was performed by adding these agents to the primary ACR50 network. Incorporation of these additional nodes had minimal effect on the estimated treatment effects of bimekizumab and secukinumab, which remained essentially unchanged. In this extended network, sonelokimab 120 mg with induction dosing showed an OR of 3.34 (95% CI 1.28–8.71) versus placebo, whereas izokibep 80 mg yielded an OR of 6.71 (2.45–18.36) versus placebo. Given the phase 2 nature of these trials, small sample sizes, and sparse data, these estimates were considered hypothesis-generating. No formal treatment rankings were computed for sonelokimab or izokibep, and the certainty of evidence for this exploratory comparison was rated very low.

### 3.7 Safety

Safety results are summarized in **Supplementary Figure 8** and **Supplementary Table 9**. In the network meta-analysis of serious adverse events (SAEs) at Weeks 12–16, no active treatment differed significantly from placebo; all 95% CIs for ORs included unity. The point estimate for secukinumab 300 mg SC was below unity, but this finding should be interpreted cautiously because of the low event rate and imprecision. Discontinuations due to adverse events were similar across all treatment arms, with no clear signal of increased withdrawal for any IL-17 inhibitor compared with placebo or adalimumab.

The risk of any infection was not significantly increased for most IL-17 inhibitors relative to placebo. However, bimekizumab 160 mg SC was associated with a modest increase in any infection risk, although the confidence interval was close to the null (OR 1.82, 95% CI 1.02–3.25 vs placebo). Candida infections occurred more frequently with IL-17 inhibitors, particularly with bimekizumab, consistent with the recognized IL-17 biology/class safety profile. Using the Peto method, the pooled OR for Candida infection with bimekizumab 160 mg versus placebo was 3.58 (95% CI 1.50–8.53), based on three trials.

New or worsening inflammatory bowel disease (IBD) events were rare and therefore unsuitable for network meta-analysis. Across all studies, only one IBD event occurred in a placebo arm (Baraliakos 2020), while total events among active treatments were bimekizumab n = 2, secukinumab n = 3, and ixekizumab n = 0. These IBD events are presented descriptively in **Supplementary Figure 8** and **Supplementary Table 9**, without formal indirect comparisons.

### 3.8 Sensitivity analyses

Ten sensitivity analyses were conducted for the primary ACR50 outcome to assess robustness of the findings (**Supplementary Table 10**). Across all scenarios, the OR for bimekizumab 160 mg SC versus placebo remained statistically significant. The base-case OR of 7.04 (5.06–9.81) persisted when the largest bimekizumab trial (BE OPTIMAL) was excluded, with an OR of 9.36 (5.06–17.31), and when key secukinumab or ixekizumab trials were removed. For example, exclusion of FUTURE 5 yielded an OR of 7.09 (4.98–10.08), and exclusion of SPIRIT-P1 yielded an OR of 7.07 (4.85–10.31).

Alternative modelling assumptions also had minimal impact on the primary estimates. Collapsing different secukinumab dose nodes, including 150 mg with and without loading, 300 mg, and intravenous secukinumab, or aggregating all secukinumab doses into a single drug-class node produced broadly similar estimates. Fixed-effect and random-effects models generated nearly identical ORs for the primary analysis, reflecting the negligible heterogeneity. Leave-one-out analyses showed that no individual study exerted undue influence on the network estimates. Overall, these sensitivity analyses indicated that the primary efficacy findings were robust to the tested assumptions, although treatment rankings varied across some scenarios.

### 3.9 Subgroup analyses

Subgroup analyses by prior biologic exposure are presented in **Supplementary Figure 11**. In the biologic DMARD-naïve (bDMARD-naïve) subgroup, based on two trials (SPIRIT-P1 and BE OPTIMAL), the magnitude of treatment effect was broadly similar to that in the overall population. In this subgroup, bimekizumab 160 mg SC had an OR of 6.90 (95% CI 4.59–10.39) versus placebo, whereas ixekizumab Q2W had an OR of 11.95 (6.16–23.16).

For patients with TNFi-inadequate response (TNFi-IR), data were available from only two trials (BE COMPLETE and SPIRIT-P2), which studied different active treatments and therefore could not be incorporated into a single connected network. As a result, formal network meta-analysis was not feasible for the TNFi-IR subgroup, and only descriptive pairwise comparisons are reported in **Supplementary Appendix S11**. No clear subgroup divergence by prior biologic exposure was observed, but the small number of studies per subgroup and wide confidence intervals limit the ability to draw firm subgroup conclusions.

### 3.10 Small-study effects

Potential small-study effects were evaluated using comparison-adjusted funnel plots and Egger’s test (**Supplementary Figure 12**). The funnel plot for the primary ACR50 network appeared visually symmetric. Egger’s test was exploratory because only eight studies were included. Egger’s test yielded an intercept of –0.31 (standard error 0.31), with a p-value of 0.321, and did not detect evidence of small-study effects. However, because these analyses involved only eight studies, the power to detect asymmetry was limited.

### 3.11 Certainty of evidence (GRADE/CINeMA)

Certainty of evidence was assessed using GRADE adapted for network meta-analysis alongside the CINeMA framework, with results summarized in **Tables 2A**, **2B**. Across outcome-level analyses, certainty ratings ranged from very low to high. High-certainty evidence was observed for the primary ACR50 network and PASI90, while several secondary efficacy outcomes, including ACR70, MDA, ACR20, and Week-24 efficacy, were graded as moderate certainty. HAQ-DI and most safety outcomes were graded as low certainty, reflecting heterogeneity, imprecision, short follow-up for harms, or sparse events. New or worsening IBD was graded as very low certainty because events were very rare and no meaningful comparative meta-analysis was possible.

**Table 2A:** Certainty of evidence using GRADE/CINeMA - efficacy outcomes.

Outcome / analysis	Evidence base	Key result	Risk of bias	Inconsistency / coherence	Indirectness	Imprecision	Other / reporting	Overall certainty	Short interpretation
ACR50 wk12-16 Primary NMA	8 RCTs 10 nodes	All active > placebo; OR 4.57-11.07; I2=0%	Not serious	Not serious; coherent network	Not serious	Not serious	No serious concern	High	Increases ACR50 with high confidence.
PASI90 wk12-16 Skin response	RCT NMA PASI subset	Large benefit; OR 5.04-30.93; I2=36%	Not serious	Not serious; same direction	Not serious	Not serious for direction	Large effect supports confidence	High	Markedly improves PASI90.
ACR70 wk12-16 Stringent joint response	RCT NMA	All active > placebo; OR 4.57-12.02; I2=35.8%	Not serious	Not serious / minor	Not serious	Serious for ranking only	No serious concern	Moderate	Probably improves ACR70.
Minimal disease activity wk12-16	RCT NMA	OR 3.30-5.96 vs placebo; I2=26%	Not serious	Not serious	Not serious	Serious; fewer events/nodes	No serious concern	Moderate	Probably increases MDA.
ACR20 wk12-16 Secondary NMA	RCT NMA	All active > placebo; OR 3.23-6.66; I2=66.9%	Not serious	Serious heterogeneity	Not serious	Not serious for direction	No serious concern	Moderate	Probably improves ACR20.
Week-24 efficacy ACR networks	Restricted RCT network	Benefit retained; IXE Q2W ACR50 OR 8.25 (2.58-26.33)	Not serious	Some heterogeneity	Not serious	Serious; restricted network	No serious concern	Moderate	Probably maintains short-term efficacy.
HAQ-DI / function wk12-16	RCT NMA	SMD about -0.54 to -0.82 for selected agents; I2=80.5%	Not serious	Serious heterogeneity	Not serious	Serious; wide CIs	No serious concern	Low	May improve physical function.

Colors indicate GRADE/CINeMA certainty levels.

**Table 2B:** Certainty of evidence using GRADE/CINeMA - safety, longer-term, and exploratory outcomes.

Outcome / analysis	Evidence base	Key result	Risk of bias	Inconsistency / coherence	Indirectness	Imprecision	Other / reporting	Overall certainty	Short interpretation
Serious adverse events wk12-24	Safety NMA	No clear increase vs placebo; most CIs include 1	Not serious	Not serious	Serious; short follow-up	Serious; sparse events	Harms reporting cautious	Low	May not increase SAEs; uncertain.
Discontinuation due to AEs wk12-24	Safety NMA	Withdrawals similar across active arms and placebo	Not serious	Not serious	Serious; short window	Serious; few events	No serious concern	Low	May have similar AE withdrawals.
Any infection wk12-24	Safety NMA	Most similar; bimekizumab OR 1.82 (1.02-3.25)	Not serious	Not serious	Serious; short follow-up	Serious; modest signal	Class-effect plausible	Low	May increase infections modestly for bimekizumab.
Candida infection wk12-24	3 trials 22 events	Bimekizumab Peto OR 3.58 (1.50-8.53)	Not serious	Not serious; I2=0%	Serious; short safety window	Serious; rare events	Biologically plausible	Low	May increase Candida infections.
Week-52 active-comparator descriptive	EXCEED + SPIRIT-H2H	Only 2 active-comparator datasets; no NMA	Some concerns	Not estimable	Serious; limited comparators	Serious; few trials	Descriptive only	Low	Long-term comparative evidence is limited.
New/worsening IBD wk12-24	Descriptive safety	Very rare: BKZ 2, SEC 3, IXE 0 active events	Some concerns	Not estimable	Serious; rare/long-term harm	Very serious; too sparse	No NMA possible	Very low	Comparative IBD safety is very uncertain.
Exploratory ACR50 sonelokimab/izokibep	Phase 2 small trials	Sonelokimab OR 3.34; izokibep OR 6.71 vs placebo	Serious; early-phase data	Not serious / sparse	Serious; exploratory nodes	Serious; small samples	Hypothesis-generating	Very low	Only exploratory; no firm ranking.

ACR, American College of Rheumatology response; AE, adverse event; CI, confidence interval; CINeMA, Confidence in Network Meta-Analysis; HAQ-DI, Health Assessment Questionnaire Disability Index; IBD, inflammatory bowel disease; MDA, minimal disease activity; NMA, network meta-analysis; OR, odds ratio; PASI, Psoriasis Area and Severity Index; RCT, randomized controlled trial; SAE, serious adverse event; SMD, standardized mean difference.

Colors indicate GRADE/CINeMA certainty levels.

Within the primary network, secukinumab 300 mg and 150 mg SC were generally supported by moderate certainty in key regimen-level summaries, whereas bimekizumab 160 mg SC was generally judged as low certainty, reflecting downgrades for risk of bias and concerns about reporting bias. Evidence for intravenous secukinumab was rated very low, reflecting imprecision, limited sample size, and indirectness. Evidence for the exploratory sonelokimab and izokibep analysis was also rated very low because the estimates were based on early-phase data, small trials, and exploratory network placement.

### 3.12 Benefit–risk composite

An exploratory benefit–risk assessment combined P-score/SUCRA-equivalent scores across efficacy and safety domains into a composite score, as detailed in **Supplementary Table 13**. The composite included ACR50, PASI90, and MDA as efficacy components, alongside inverted scores for serious adverse events and Candida infections as safety components, under three pre-specified weighting schemes: balanced, efficacy-first, and safety-first. Under the balanced weighting and the efficacy-first scenario, bimekizumab 160 mg with loading and bimekizumab 160 mg without loading obtained the highest composite scores (balanced: 61.8 and 60.6; efficacy-first: 66.2 and 60.9, respectively), indicating favorable combined performance on multidomain efficacy and tolerability despite the infection and Candida signal.

When safety was prioritized (Scenario 3), secukinumab 300 mg SC attained the highest composite score (64.6), consistent with its more favorable SAE profile and lower Candida rates compared with dual IL-17A/F inhibition. Placebo ranked last in all benefit–risk scenarios. This composite analysis was exploratory and should be interpreted with caution, particularly given the low certainty of evidence for bimekizumab and the inherent limitations of combining disparate outcomes into a single score.

### 3.13 Week-24 and longer-term outcomes

Longer-term efficacy was explored in Week-24 and Week-52 analyses. Week-24 network meta-analyses were restricted to trials that maintained a placebo or control arm through Week 24, resulting in a smaller and less connected network. Within this restricted Week-24 network, ixekizumab Q2W had the largest estimated effect versus placebo for ACR50, with an OR of 8.25 (95% CI 2.58–26.33), although between-study heterogeneity increased compared with the Week 12–16 analyses.

At Week 52, only two head-to-head trials, EXCEED and SPIRIT-H2H, provided suitable comparative data, precluding network meta-analysis. Descriptive pairwise analyses from these trials suggested broadly comparable long-term efficacy between the active comparators studied, without a clear separation in joint or skin outcomes over one year, based on limited head-to-head evidence.

## 4 Discussion

This network meta-analysis found that IL-17 pathway inhibitors were consistently superior to placebo for ACR50 at Weeks 12–16 in adults with active psoriatic arthritis. Ixekizumab Q2W had the highest P-score for the primary ACR50 outcome, while bimekizumab, ixekizumab, and secukinumab showed clinically relevant improvements across joint, skin, functional, and multidomain outcomes. However, most active-treatment comparisons had overlapping confidence intervals, and treatment rankings should therefore be interpreted as supportive rather than definitive evidence of superiority. A clinically relevant caveat is that the high ranking of ixekizumab Q2W reflects induction-period dosing in the included trials rather than the licensed Q4W maintenance schedule; this distinction should be considered when translating short-term rankings into long-term practice. The primary ACR50 network showed negligible heterogeneity and no evidence of important incoherence, although heterogeneity was more pronounced for HAQ-DI and some secondary outcomes.

These findings are consistent with previous systematic reviews and network meta-analyses showing that biologic therapies, including IL-17 inhibitors, improve joint and skin outcomes in active PsA. Earlier work, including the network meta-analysis by Cawson et al. (12), established the superiority of biologic therapies over placebo but was based on an older evidence base with fewer targeted agents and limited head-to-head data. Naik et al. (13) showed that IL-17 pathway inhibitors improved ACR20 responses and had acceptable short-term safety, but the analysis included fewer early trials and did not evaluate newer agents or more stringent multidomain endpoints. More recently, Gao et al. (14) confirmed the efficacy of IL-17, IL-12/23, and IL-23 inhibitors across joint and skin domains, while also identifying class-relevant safety signals such as Candida infections. The present analysis extends this literature by focusing specifically on IL-17 pathway inhibition, evaluating clinically relevant dose-level regimens, incorporating bimekizumab and exploratory phase 2 agents, and integrating sensitivity, subgroup, Bayesian, benefit–risk, and CINeMA/GRADE assessments. Cross-class network meta-analyses are useful for deciding whether IL-17 inhibition should be selected among targeted therapies, whereas the present analysis provides a focused within-class evidence map once IL-17 inhibition is considered clinically appropriate.

The pattern of efficacy observed in this analysis is biologically plausible. IL-17A and IL-17F contribute to psoriatic inflammation across synovial, entheseal, cutaneous, and bone-related tissues, supporting the broad clinical effects observed across ACR, PASI90, MDA, and HAQ-DI outcomes (7, 8). Selective IL-17A inhibitors such as secukinumab and ixekizumab have already established the clinical relevance of this pathway, while dual IL-17A/F inhibition with bimekizumab provides a mechanistically distinct approach because IL-17F has overlapping pro-inflammatory activity with IL-17A (9, 35). This provides a biologically plausible explanation for the favorable rankings observed for bimekizumab across several efficacy outcomes, although the available indirect comparisons do not establish definitive superiority over selective IL-17A inhibition.

Accordingly, the novelty of this study should be interpreted as incremental and clinically focused rather than as the first demonstration that IL-17 inhibition is effective in PsA. Broad cross-class NMAs remain more informative when clinicians are deciding between IL-17 inhibition and other mechanisms, such as TNF, IL-23, IL-12/23, or Janus kinase inhibition. In contrast, the present study addresses a narrower decision point: how the available evidence differs among individual IL-17 pathway agents and regimens after this pathway has been selected as an appropriate therapeutic option. The regimen-level network structure, harmonized ACR50 assessment window, inclusion of dual IL-17A/F inhibition and emerging agents, and simultaneous evaluation of joint, skin, multidomain, functional, and class-relevant safety outcomes provide a more detailed within-class evidence map. Nevertheless, because most comparisons between active treatments were indirect, this additional resolution should be regarded as decision support rather than evidence of a definitive therapeutic hierarchy.

Safety findings were broadly consistent with the known role of IL-17 in mucosal host defence. Serious adverse events and discontinuations due to adverse events were not clearly increased compared with placebo during the double-blind periods analyzed. However, bimekizumab was associated with a modest increase in any infection risk and a higher Candida signal, consistent with recognized IL-17 pathway biology and the role of IL-17 signaling in antifungal defence (10, 36). Most importantly, these safety findings should be interpreted in the context of short double-blind follow-up and sparse event counts. New or worsening inflammatory bowel disease events were rare and unsuitable for comparative network meta-analysis, so gastrointestinal safety remains uncertain, particularly over longer follow-up.

Translation of these findings into practice requires a phenotype-first interpretation rather than selection based solely on the numerical treatment rankings. In patients with active peripheral arthritis accompanied by substantial plaque psoriasis, agents showing strong effects across both joint and skin outcomes may be particularly attractive. In the present analysis, ixekizumab and bimekizumab produced favorable estimates across ACR and PASI90 outcomes, while bimekizumab also performed strongly for minimal disease activity. Consequently, bimekizumab may be a reasonable option for patients requiring simultaneous control of musculoskeletal and severe cutaneous disease, particularly when there is no history of recurrent mucocutaneous Candida infection or other important infection-related concern. However, the apparent advantage should not be interpreted as proven superiority because most active-treatment comparisons were indirect, confidence intervals overlapped, and certainty for some bimekizumab comparisons was lower than for the more established IL-17A inhibitors. The high short-term ACR50 ranking of ixekizumab Q2W should also be interpreted in the context of induction-period dosing and should not be extrapolated directly to long-term maintenance selection.

Safety history may shift this balance. In patients with recurrent oral, esophageal, cutaneous, or vulvovaginal candidiasis, the higher Candida signal observed with dual IL-17A/F inhibition may reduce the attractiveness of bimekizumab despite its favorable multidomain efficacy estimates. In such patients, a selective IL-17A inhibitor or an agent outside the IL-17 pathway may be more appropriate, depending on the dominant PsA domains and previous treatment history. This interpretation should remain cautious because Candida events were uncommon, follow-up was short, and the current evidence does not establish that selective IL-17A inhibitors are free from this class-related risk. Before initiating treatment, clinicians should therefore consider previous fungal infection, other factors that may predispose to infection, and the patient’s willingness to undergo monitoring and seek early treatment if symptoms of candidiasis develop.

Gastrointestinal comorbidity represents a separate and clinically important decision point. In patients with active inflammatory bowel disease, or a convincing history of treatment-related worsening of intestinal inflammation, the low number of IBD events in this review should not be interpreted as evidence of gastrointestinal safety. Current therapeutic recommendations emphasize that coexisting IBD should influence the selected mechanism of action, and regulatory guidance advises avoidance of bimekizumab in patients with active IBD (6, 11). In this setting, a therapy with demonstrated efficacy across both PsA and IBD may be preferable to an IL-17 inhibitor. Because IBD events in the included trials were extremely rare and could not be analyzed comparatively, the present NMA cannot determine whether meaningful gastrointestinal safety differences exist among bimekizumab, secukinumab, and ixekizumab.

Prior treatment exposure and the predominant musculoskeletal phenotype should also guide interpretation. Among patients with inadequate response or intolerance to a TNF inhibitor, bimekizumab and ixekizumab are supported by dedicated phase 3 placebo-controlled evidence from BE COMPLETE and SPIRIT-P2, respectively (19, 20). However, these trials formed a disconnected network, preventing a valid comparative estimate between the two agents; therefore, treatment selection in TNFi-inadequate responders should be guided by skin involvement, previous adverse events, infection and IBD history, dosing preferences, and access rather than by the overall network ranking. Similarly, in patients with predominant axial manifestations, domain-specific evidence, such as the MAXIMISE trial of secukinumab, should receive greater weight than rankings based primarily on peripheral ACR responses (21). For biologic-naïve patients without a dominant extra-articular comorbidity, the overlapping active-treatment estimates suggest that injection frequency, loading requirements, concomitant therapy, availability, cost, and patient preference may reasonably become decisive considerations. These scenarios reinforce that P-scores should complement, rather than replace, domain-based assessment and shared decision-making.

Strengths of this study include its focused IL-17 pathway scope, updated evidence base, dose-level network structure, and evaluation of multidomain efficacy alongside class-relevant safety outcomes. The analysis incorporated sensitivity analyses, subgroup analyses by prior biologic exposure, Bayesian validation, treatment ranking, CINeMA/GRADE certainty assessment, and an exploratory benefit–risk model. The primary ACR50 findings were robust across tested assumptions, including exclusion of key large trials, alternative dose-node assumptions, fixed-effect modelling, inclusion of exploratory agents, and leave-one-out analyses.

Several limitations should be considered. First, most comparisons between active IL-17 inhibitors were indirect, and direct head-to-head evidence within the IL-17 class remains limited. Moreover, treatment-level results were derived from trial populations and aggregate data and could not account for individual combinations of skin severity, recurrent Candida infection, gastrointestinal comorbidity, treatment adherence, access, or patient preference; the clinical scenarios discussed above should therefore be considered interpretative applications of the evidence rather than directly tested treatment-selection algorithms. The review was intentionally restricted to ligand-targeting IL-17 therapies and therefore did not evaluate brodalumab, which blocks IL-17RA and has been investigated in phase 2 and phase 3 PsA trials. Consequently, the findings should not be generalized to all forms of IL-17 pathway blockade, particularly receptor-targeting therapy. Second, the double-blind follow-up periods were short, limiting inference about long-term safety, sustained efficacy, radiographic outcomes, and rare adverse events such as IBD. Third, some subgroup analyses, particularly among TNFi-inadequate responders, were limited by disconnected networks and small numbers of trials. In addition, pre-specified subgroup analyses by methotrexate use and baseline disease activity could not be performed because trial-level reporting was insufficiently consistent across studies. Fourth, HAQ-DI and some secondary outcomes showed greater heterogeneity than the primary ACR50 analysis, which reduces confidence in precise comparative estimates for functional outcomes. Fifth, sonelokimab and izokibep were supported only by early-phase evidence and very low certainty, so their results should be considered hypothesis-generating. Finally, the benefit–risk composite combined heterogeneous efficacy and safety components using relative rankings rather than absolute event rates, and therefore should not be interpreted as a validated clinical decision rule.

Future research should prioritize direct head-to-head randomized controlled trials comparing dual IL-17A/F blockade with selective IL-17A inhibition. Longer-term extension studies and post-marketing data are needed to better define sustained efficacy, radiographic progression, treatment persistence, and rare safety outcomes such as serious infections and inflammatory bowel disease. Further investigation into patient-level predictors of response, including prior biologic exposure, clinical phenotype, comorbidity burden, and biomarker-driven stratification, would support more individualized treatment selection. Real-world registries will also be important to validate comparative effectiveness and safety in routine clinical practice over extended follow-up.

## 5 Conclusion

IL-17 pathway inhibitors are effective treatments for active psoriatic arthritis, with consistent short-term benefits across joint and skin outcomes compared with placebo. Differences in treatment rankings suggest possible variation between agents, but definitive superiority between active treatments remains uncertain because most comparisons are indirect and confidence intervals overlap. Candida infection risk, particularly with dual IL-17A/F inhibition, should be considered when selecting therapy. Certainty was higher for short-term efficacy outcomes and lower for safety, functional, long-term, and exploratory analyses. Treatment choice should therefore be individualized rather than determined by rankings alone. Patients with substantial concomitant skin disease may favor regimens with strong joint and PASI90 effects, whereas recurrent Candida infection, active or previous IBD, prior biologic failure, predominant axial disease, dosing burden, access, and patient preference may shift selection toward another IL-17 inhibitor or a different therapeutic mechanism.

## Data Availability

The datasets used and/or analysed during the current study and the analytic code are available from the corresponding author upon reasonable request.

## Glossary

ACR: American College of Rheumatology
ACR20: ≥20% improvement in American College of Rheumatology response criteria
ACR50: ≥50% improvement in American College of Rheumatology response criteria
ACR70: ≥70% improvement in American College of Rheumatology response criteria
AE: Adverse event
bDMARD: Biologic disease-modifying antirheumatic drug
CASPAR: Classification Criteria for Psoriatic Arthritis
CENTRAL: Cochrane Central Register of Controlled Trials
CI: Confidence interval
CINeMA: Confidence in Network Meta-Analysis
CrI: Credible interval
DMARD: Disease-modifying antirheumatic drug
EULAR: European Alliance of Associations for Rheumatology
GRADE: Grading of Recommendations Assessment, Development and Evaluation
GRAPPA: Group for Research and Assessment of Psoriasis and Psoriatic Arthritis
HAQ-DI: Health Assessment Questionnaire Disability Index
IBD: Inflammatory bowel disease
IL: Interleukin
IL-17A/F: Interleukin-17A and interleukin-17F
IV: Intravenous
JAGS: Just Another Gibbs Sampler
MCMC: Markov chain Monte Carlo
MDA: Minimal disease activity
MeSH: Medical Subject Headings
NMA: Network meta-analysis
OR: Odds ratio
PASI90: ≥90% reduction in Psoriasis Area and Severity Index
PRISMA: Preferred Reporting Items for Systematic Reviews and Meta-Analyses
PROSPERO: International Prospective Register of Systematic Reviews
PsA: Psoriatic arthritis
Q2W: Every 2 weeks
Q4W: Every 4 weeks
RCT: Randomized controlled trial
RoB 2.0: Revised Cochrane Risk of Bias tool for randomized trials
SAE: Serious adverse event
SC: Subcutaneous
SD: Standard deviation
SE: Standard error
SMD: Standardized mean difference
SUCRA: Surface under the cumulative ranking curve
TNF: Tumor necrosis factor
TNFi: Tumor necrosis factor inhibitor.

## References

[1] Azuaga AB Ramírez J Cañete JD . Psoriatic arthritis: pathogenesis and targeted therapies. Int J Mol Sci. (2023) 24:4901. doi: 10.3390/ijms24054901 36902329 PMC10003101

[2] Lembke S Macfarlane GJ Jones GT . The worldwide prevalence of psoriatic arthritis—a systematic review and meta-analysis. Rheumatology. (2024) 63:3211–20. doi: 10.1093/rheumatology/keae198 38530786 PMC11637478

[3] Ogdie A Hur P Liu M Rebello S McLean RR Dube B . Effect of multidomain disease presentations on patients with psoriatic arthritis in the Corrona Psoriatic Arthritis/Spondyloarthritis Registry. J Rheumatol. (2020) 48:698–706. doi: 10.3899/jrheum.200371 33004532

[4] James L Hailey LH Suribhatla R McGagh D Amarnani R Bundy CE . The impact of psoriatic arthritis on quality of life: a systematic review. Ther Adv Musculoskeletal Dis. (2024) 16:1759720X241295920. doi: 10.1177/1759720x241295920 39717741 PMC11664531

[5] Coates LC Soriano ER Bertheussen H Duffin KC Campanholo CB Chau J . Group for Research and Assessment of Psoriasis and Psoriatic Arthritis (GRAPPA): updated treatment recommendations for psoriatic arthritis 2021. Nat Rev Rheumatol. (2022) 18:465–79. doi: 10.1038/s41584-022-00798-0 35761070 PMC9244095

[6] Gossec L Kerschbaumer A Ferreira RJO Aletaha D Baraliakos X Bertheussen H . EULAR recommendations for the management of psoriatic arthritis with pharmacological therapies: 2023 update. Ann Rheumatic Dis. (2024) 83:706–19. doi: 10.1136/ard-2024-225531 38499325 PMC11103320

[7] Blauvelt A Chiricozzi A . The immunologic role of IL-17 in psoriasis and psoriatic arthritis pathogenesis. Clin Rev Allergy Immunol. (2018) 55:379–90. doi: 10.1007/s12016-018-8702-3 30109481 PMC6244934

[8] Wang EA Suzuki E Maverakis E Adamopoulos IE . Targeting IL-17 in psoriatic arthritis. Eur J Rheumatol. (2017) 4:272–7. doi: 10.5152/eurjrheum.2017.17037 29308283 PMC5741341

[9] Glatt S Baeten D Baker T Griffiths M Ionescu L Lawson ADG . Dual IL-17A and IL-17F neutralisation by bimekizumab in psoriatic arthritis: evidence from preclinical experiments and a randomised placebo-controlled clinical trial that IL-17F contributes to human chronic tissue inflammation. Ann Rheumatic Dis. (2017) 77:523–32. doi: 10.1136/annrheumdis-2017-212127 29275332 PMC5890624

[10] Mengesha B Conti H . The role of IL-17 in protection against mucosal candida infections. J Fungi. (2017) 3:52. doi: 10.3390/jof3040052 29371568 PMC5753154

[11] Fauny M Moulin D D’Amico F Netter P Petitpain N Arnone D . Paradoxical gastrointestinal effects of interleukin-17 blockers. Ann Rheumatic Dis. (2020) 79:1132–8. doi: 10.1136/annrheumdis-2020-217927 32719044

[12] Cawson MR Mitchell SA Knight C Wildey H Spurden D Bird A . Systematic review, network meta-analysis and economic evaluation of biological therapy for the management of active psoriatic arthritis. BMC Musculoskeletal Disord. (2014) 15:26. doi: 10.1186/1471-2474-15-26 24444034 PMC3903562

[13] Naik GS Ming WK Magodoro IM Akinwunmi B Dar S Poulsen HE . TH17 inhibitors in active psoriatic arthritis: A systematic review and meta-analysis of randomized controlled clinical trials. Dermatology. (2017) 233:366–77. doi: 10.1159/000484520 29258093

[14] Gao S Xie X Fan L Yu L . Efficacy and safety of IL-17, IL-12/23, and IL-23 inhibitors for psoriatic arthritis: a network meta-analysis of randomized controlled trials. Front Immunol. (2025) 16:1654343. doi: 10.3389/fimmu.2025.1654343 41050680 PMC12489988

[15] Page MJ McKenzie JE Bossuyt PM Boutron I Hoffmann TC Mulrow CD . The PRISMA 2020 statement: an updated guideline for reporting systematic reviews. BMJ. (2021) 372:n71. doi: 10.1136/bmj.n71 33782057 PMC8005924

[16] Abbas A Hefnawy MT Negida A . Meta-analysis accelerator: a comprehensive tool for statistical data conversion in systematic reviews with meta-analysis. BMC Med Res Methodol. (2024) 24:243. doi: 10.1186/s12874-024-02356-6 39425031 PMC11487830

[17] Sterne JC Savović J Page MJ Elbers RG Blencowe NS Boutron I . RoB 2: a revised tool for assessing risk of bias in randomised trials. BMJ. (2019) 366:l4898. doi: 10.1136/bmj.l4898 31462531

[18] McGuinness LA Higgins JPT . Risk‐of‐bias VISualization (robvis): An R package and Shiny web app for visualizing risk‐of‐bias assessments. Res Synth Methods. (2020) 12:55–61. doi: 10.1002/jrsm.1411 32336025

[19] Nash P Kirkham B Okada M Rahman P Combe B Burmester G . Ixekizumab for the treatment of patients with active psoriatic arthritis and an inadequate response to tumour necrosis factor inhibitors: results from the 24-week randomised, double-blind, placebo-controlled period of the SPIRIT-P2 phase 3 trial. Lancet. (2017) 389:2317–27. doi: 10.1016/s0140-6736(17)31429-0 28551073

[20] Merola JF Landewé R McInnes IB Mease PJ Ritchlin CT Tanaka Y . Bimekizumab in patients with active psoriatic arthritis and previous inadequate response or intolerance to tumour necrosis factor-α inhibitors: a randomised, double-blind, placebo-controlled, phase 3 trial (BE COMPLETE). Lancet. (2022) 401:38–48. doi: 10.1016/s0140-6736(22)02303-0 36495881

[21] Baraliakos X Gossec L Pournara E Jeka S Mera-Varela A D’Angelo S . Secukinumab in patients with psoriatic arthritis and axial manifestations: results from the double-blind, randomised, phase 3 MAXIMISE trial. Ann Rheumatic Dis. (2020) 80:582–90. doi: 10.1136/annrheumdis-2020-218808 33334727 PMC8053347

[22] Kivitz AJ Nash P Tahir H Everding A Mann H Kaszuba A . Efficacy and safety of subcutaneous secukinumab 150 mg with or without loading regimen in psoriatic arthritis: results from the FUTURE 4 study. Rheumatol Ther. (2019) 6:393–407. doi: 10.1007/s40744-019-0163-5 31228101 PMC6702584

[23] Kivitz A Sedova L Churchill M Kotha R Singhal A Torres A . Efficacy and safety of intravenous secukinumab for the treatment of active psoriatic arthritis: results from a randomized, Placebo‐Controlled Phase 3 study. Arthritis Rheumatol. (2024) 77:171–9. doi: 10.1002/art.42997 39300596 PMC11782105

[24] Mease P Van Der Heijde D Landewé R Mpofu S Rahman P Tahir H . Secukinumab improves active psoriatic arthritis symptoms and inhibits radiographic progression: primary results from the randomised, double-blind, phase III FUTURE 5 study. Ann Rheumatic Dis. (2018) 77:890–7. doi: 10.1136/annrheumdis-2017-212687 29550766 PMC5965348

[25] Mease PJ McInnes IB Kirkham B Kavanaugh A Rahman P Van Der Heijde D . Secukinumab inhibition of interleukin-17A in patients with psoriatic arthritis. N Engl J Med. (2015) 373:1329–39. doi: 10.1056/nejmoa1412679 26422723

[26] McInnes IB Mease PJ Kirkham B Kavanaugh A Ritchlin CT Rahman P . Secukinumab, a human anti-interleukin-17A monoclonal antibody, in patients with psoriatic arthritis (FUTURE 2): a randomised, double-blind, placebo-controlled, phase 3 trial. Lancet. (2015) 386:1137–46. doi: 10.1016/s0140-6736(15)61134-5 26135703

[27] Nash P Mease PJ McInnes IB Rahman P Ritchlin CT Blanco R . Efficacy and safety of secukinumab administration by autoinjector in patients with psoriatic arthritis: results from a randomized, placebo-controlled trial (FUTURE 3). Arthritis Res Ther. (2018) 20:47. doi: 10.1186/s13075-018-1551-x 29544534 PMC5856314

[28] Mease PJ Van Der Heijde D Ritchlin CT Okada M Cuchacovich RS Shuler CL . Ixekizumab, an interleukin-17A specific monoclonal antibody, for the treatment of biologic-naive patients with active psoriatic arthritis: results from the 24-week randomised, double-blind, placebo-controlled and active (adalimumab)-controlled period of the phase III trial SPIRIT-P1. Ann Rheumatic Dis. (2016) 76:79–87. doi: 10.1136/annrheumdis-2016-209709 27553214 PMC5264219

[29] Smolen JS Sebba A Ruderman EM Schulze-Koops H Sapin C Gellett AM . Efficacy and safety of ixekizumab with or without methotrexate in biologic-naïve patients with psoriatic arthritis: 52-week results from SPIRIT-H2H study. Rheumatol Ther. (2020) 7:1021–35. doi: 10.1007/s40744-020-00250-3 33200394 PMC7695764

[30] Ritchlin CT Kavanaugh A Merola JF Schett G Scher JU Warren RB . Bimekizumab in patients with active psoriatic arthritis: results from a 48-week, randomised, double-blind, placebo-controlled, dose-ranging phase 2b trial. Lancet. (2020) 395:427–40. doi: 10.1016/s0140-6736(19)33161-7 32035552

[31] McInnes IB Asahina A Coates LC Landewé R Merola JF Ritchlin CT . Bimekizumab in patients with psoriatic arthritis, naive to biologic treatment: a randomised, double-blind, placebo-controlled, phase 3 trial (BE OPTIMAL). Lancet. (2022) 401:25–37. doi: 10.1016/s0140-6736(22)02302-9 36493791

[32] Gottlieb A Merola J Reich K Behrens F Nash P Griffiths C . Efficacy of secukinumab and adalimumab in patients with psoriatic arthritis and concomitant moderate‐to‐severe plaque psoriasis: results from EXCEED, a randomized, double‐blind head‐to‐head monotherapy study. Br J Dermatol. (2021) 185:1124–34. doi: 10.1111/bjd.20413 33913511 PMC9291158

[33] McInnes IB Coates LC Mease PJ Ogdie A Kavanaugh A Eder L . Sonelokimab, an IL-17A/IL-17F-inhibiting nanobody for active psoriatic arthritis: a randomized, placebo-controlled phase 2 trial. Nat Med. (2025) 31:4160–71. doi: 10.1038/s41591-025-03971-6 41053449 PMC12705426

[34] Taylor PC Mease PJ De Vlam K Mpofu S Wetzel D Stevens AM . Efficacy and safety of izokibep in patients with active psoriatic arthritis: a randomised, double-blind, placebo-controlled, phase 2 study. Ann Rheumatic Dis. (2025) 84:979–91. doi: 10.1016/j.ard.2025.02.019 40455082

[35] Ali Z Matthews R Al-Janabi A Warren RB . Bimekizumab: a dual IL-17A and IL-17F inhibitor for the treatment of psoriasis and psoriatic arthritis. Expert Rev Clin Immunol. (2021) 17:1073–81. doi: 10.1080/1744666x.2021.1967748 34384327

[36] Conti HR Gaffen SL . IL-17–mediated immunity to the opportunistic fungal pathogen Candida albicans. J Immunol. (2015) 195:780–8. doi: 10.4049/jimmunol.1500909 PMC4507294 26188072

