# Supplementary material for: IL-17 ligand-targeting inhibitors in psoriatic arthritis: a focused systematic review and network meta-analysis of efficacy, safety, and certainty of evidence
Source: Front Immunol. 2026 Aug 13;17:1893092. doi: 10.3389/fimmu.2026.1893092 (PMC13518361; doi:10.3389/fimmu.2026.1893092)
Supplement: Supplementary file 1 [file SupplementaryFile1.docx]

**SUPPLEMENTARY MATERIAL**

**Contents of the Supplementary Appendix**

This file consolidates all supplementary tables and figures into a single submission-ready Word document. Figure and table titles have been standardized, while the scientific content from the uploaded supplementary files has been preserved.

| **Item** | **Title** | **Format** |
| --- | --- | --- |
| **S1** | S1. PRISMA 2020 Checklist | Table |
| **S2** | S2. Full Electronic Search Strategy | Table |
| **S3** | S3. Characteristics of Included Randomized Controlled Trials | Table |
| **S4** | Figures S4A-B. Risk-of-Bias Assessment | Figure |
| **S5** | S5. Treatment Ranking for ACR50 at Weeks 12-16 | Figure |
| **S6** | S6. Secondary Efficacy Network Meta-analyses at Weeks 12-16 | Figure |
| **S7** | S7. Node-splitting: Direct Versus Indirect Evidence | Figure |
| **S8** | S8. Safety Network Meta-analyses | Figure |
| **S9** | S9. Safety Summary From Included Studies | Table |
| **S10** | S10. Sensitivity Analyses for ACR50 Network Estimates | Table |
| **S11** | S11. Subgroup Analyses by Prior Biologic Exposure | Figure |
| **S12** | S12. Comparison-adjusted Funnel Plot for ACR50 | Figure |
| **S13** | S13. Exploratory Benefit-risk Composite Analysis | Table |

**S1**

**Supplementary Table S1. PRISMA 2020 Checklist**

Supplementary Table S1. PRISMA 2020 checklist.

| **Section and topic** | **Item #** | **Checklist item** | **Page number** |
| --- | --- | --- | --- |
| Title - Title | 1 | Identify the report as a systematic review. | 1 |
| Abstract - Abstract | 2 | See the PRISMA 2020 for Abstracts checklist. | 3,4 |
| Introduction - Rationale | 3 | Describe the rationale for the review in the context of existing knowledge. | 4,5 |
| Introduction - Objectives | 4 | Provide an explicit statement of the objective(s) or question(s) the review addresses. | 5 |
| Methods - Eligibility criteria | 5 | Specify the inclusion and exclusion criteria for the review and how studies were grouped for the syntheses. | 6,7 |
| Methods - Information sources | 6 | Specify all databases, registers, websites, organisations, reference lists and other sources searched or consulted to identify studies. Specify the date when each source was last searched or consulted. | 6 |
| Methods - Search strategy | 7 | Present the full search strategies for all databases, registers and websites, including any filters and limits used. | 6, Table 2/S2 |
| Methods - Selection process | 8 | Specify the methods used to decide whether a study met the inclusion criteria of the review, including how many reviewers screened each record and each report retrieved, whether they worked independently, and if applicable, details of automation tools used in the process. | 7,8 |
| Methods - Data collection process | 9 | Specify the methods used to collect data from reports, including how many reviewers collected data from each report, whether they worked independently, any processes for obtaining or confirming data from study investigators, and if applicable, details of automation tools used in the process. | 8 |
| Methods - Data items | 10a | List and define all outcomes for which data were sought. Specify whether all results that were compatible with each outcome domain in each study were sought (e.g. for all measures, time points, analyses), and if not, the methods used to decide which results to collect. | 7 |
| Methods - Data items | 10b | List and define all other variables for which data were sought (e.g. participant and intervention characteristics, funding sources). Describe any assumptions made about any missing or unclear information. | 8 |
| Methods - Study risk of bias assessment | 11 | Specify the methods used to assess risk of bias in the included studies, including details of the tool(s) used, how many reviewers assessed each study and whether they worked independently, and if applicable, details of automation tools used in the process. | 8,9 |
| Methods - Effect measures | 12 | Specify for each outcome the effect measure(s) (e.g. risk ratio, mean difference) used in the synthesis or presentation of results. | 9 |
| Methods - Synthesis methods | 13a | Describe the processes used to decide which studies were eligible for each synthesis (e.g. tabulating the study intervention characteristics and comparing against the planned groups for each synthesis (item #5)). | 7,9 |
| Methods - Synthesis methods | 13b | Describe any methods required to prepare the data for presentation or synthesis, such as handling of missing summary statistics, or data conversions. | 8,9 |
| Methods - Synthesis methods | 13c | Describe any methods used to tabulate or visually display results of individual studies and syntheses. | 9 |
| Methods - Synthesis methods | 13d | Describe any methods used to synthesise results and provide a rationale for the choice(s). If meta-analysis was performed, describe the model(s), method(s) to identify the presence and extent of statistical heterogeneity, and software package(s) used. | 9-12 |
| Methods - Synthesis methods | 13e | Describe any methods used to explore possible causes of heterogeneity among study results (e.g. subgroup analysis, meta-regression). | 9,10 |
| Methods - Synthesis methods | 13f | Describe any sensitivity analyses conducted to assess robustness of the synthesised results. | 10, Table S10 |
| Methods - Reporting bias assessment | 14 | Describe any methods used to assess risk of bias due to missing results in a synthesis (arising from reporting biases). | 9 |
| Methods - Certainty assessment | 15 | Describe any methods used to assess certainty (or confidence) in the body of evidence for an outcome. | 12 |
| Results - Study selection | 16a | Describe the results of the search and selection process, from the number of records identified in the search to the number of studies included in the review, ideally using a flow diagram. | 12,13, Figure 1 |
| Results - Study selection | 16b | Cite studies that might appear to meet the inclusion criteria, but which were excluded, and explain why they were excluded. | 13, Figure 1 |
| Results - Study characteristics | 17 | Cite each included study and present its characteristics. | 13; Table S3 |
| Results - Risk of bias in studies | 18 | Present assessments of risk of bias for each included study. | 13; S4A-B |
| Results - Results of individual studies | 19 | For all outcomes, present, for each study: (a) summary statistics for each group (where appropriate) and (b) an effect estimate and its precision (e.g. confidence/credible interval), ideally using structured tables or plots. | 14-19, Figure 3, Table 1, S6A-D, S8, S9 |
| Results - Results of syntheses | 20a | For each synthesis, briefly summarise the characteristics and risk of bias among contributing studies. | 13-19, S3, S4A-B |
| Results - Results of syntheses | 20b | Present results of all statistical syntheses conducted. If meta-analysis was done, present for each the summary estimate and its precision (e.g. confidence/credible interval) and measures of statistical heterogeneity. If comparing groups, describe the direction of the effect. | 14-19, Figure 3, Table 1, Table 2, S5-S8, S9, S13 |
| Results - Results of syntheses | 20c | Present results of all investigations of possible causes of heterogeneity among study results. | 17-18, S11 |
| Results - Results of syntheses | 20d | Present results of all sensitivity analyses conducted to assess the robustness of the synthesised results. | 17, S10 |
| Results - Reporting biases | 21 | Present assessments of risk of bias due to missing results (arising from reporting biases) for each synthesis assessed. | 18, S12 |
| Results - Certainty of evidence | 22 | Present assessments of certainty (or confidence) in the body of evidence for each outcome assessed. | 18-19; Table 2 |
| Discussion - Discussion | 23a | Provide a general interpretation of the results in the context of other evidence. | 20,21 |
| Discussion - Discussion | 23b | Discuss any limitations of the evidence included in the review. | 21,22 |
| Discussion - Discussion | 23c | Discuss any limitations of the review processes used. | 21,22 |
| Discussion - Discussion | 23d | Discuss implications of the results for practice, policy, and future research. | 21,22 |
| Other information - Registration and protocol | 24a | Provide registration information for the review, including register name and registration number, or state that the review was not registered. | 6 |
| Other information - Registration and protocol | 24b | Indicate where the review protocol can be accessed, or state that a protocol was not prepared. | 6 |
| Other information - Registration and protocol | 24c | Describe and explain any amendments to information provided at registration or in the protocol. | 6 |
| Other information - Support | 25 | Describe sources of financial or non-financial support for the review, and the role of the funders or sponsors in the review. | 22 |
| Other information - Competing interests | 26 | Declare any competing interests of review authors. | 22, 23 |
| Other information - Availability of data, code, and other materials | 27 | Report which of the following are publicly available and where they can be found: template data collection forms; data extracted from included studies; data used for all analyses; analytic code; any other materials used in the review. | 22 |

**S2**

**Supplementary Table S2. Full Electronic Search Strategy**

Supplementary Table S2. Full electronic search strategy by database/register.

| **Database** | **Search strategy** |
| --- | --- |
| PubMed/ MEDLINE (n = 315) | (("Arthritis, Psoriatic"[Mesh] OR "Psoriatic Arthritis"[tiab] OR "PsA"[tiab] OR "Psoriatic Arthropathy"[tiab]) AND ("Interleukin-17"[Mesh] OR "IL-17 inhibitor"[tiab] OR "Anti-IL-17"[tiab] OR "IL-17 blockade"[tiab] OR "Secukinumab"[tiab] OR "AIN457"[tiab] OR "Ixekizumab"[tiab] OR "LY2439821"[tiab] OR "Bimekizumab"[tiab] OR "UCB4940"[tiab] OR "Izokibep"[tiab] OR "Sonelokimab"[tiab]) AND ("randomized controlled trial"[Publication Type] OR "RCT"[Publication Type] OR "controlled clinical trial"[Publication Type] OR "randomized"[Title/Abstract] OR "randomised"[Title/Abstract] OR "placebo"[Title/Abstract] OR "RCT"[Title/Abstract] OR "trial"[Title/Abstract]) AND ("2010/01/01"[Date - Publication] : "3000"[Date - Publication])) |
| Scopus (n = 918) | TITLE-ABS-KEY(("Psoriatic Arthritis" OR PsA OR "Psoriatic Arthropathy") AND ("Interleukin-17" OR "IL-17 inhibitor" OR "Anti-IL-17" OR "IL-17 blockade" OR Secukinumab OR AIN457 OR Ixekizumab OR LY2439821 OR Bimekizumab OR UCB4940 OR Izokibep OR Sonelokimab) AND ("randomized controlled trial" OR "controlled clinical trial" OR randomized OR randomised OR placebo OR trial OR RCT)) AND (PUBYEAR > 2009 AND PUBYEAR < 2026) |
| Embase (n = 850 ) | ('psoriatic arthritis'/exp OR 'psoriatic arthritis':ti,ab OR 'psa':ti,ab OR 'psoriatic arthropathy':ti,ab) AND ('interleukin 17'/exp OR 'il 17 inhibitor':ti,ab OR 'anti il 17':ti,ab OR 'il 17 blockade':ti,ab OR 'secukinumab':ti,ab OR 'ain457':ti,ab OR 'ixekizumab':ti,ab OR 'ly2439821':ti,ab OR 'bimekizumab':ti,ab OR 'ucb4940':ti,ab OR 'izokibep':ti,ab OR 'sonelokimab':ti,ab) AND ('randomized controlled trial'/exp OR 'controlled clinical trial'/exp OR randomized:ti,ab OR randomised:ti,ab OR placebo:ti,ab OR trial:ti,ab OR rct:ti,ab) AND [2010-2025]/py |
| Cochrane Library (n = 116) | ([mh "Psoriatic Arthritis"] OR "Psoriatic Arthritis":ti,ab OR PsA:ti,ab OR "Psoriatic Arthropathy":ti,ab) AND ([mh "Interleukin-17"] OR "IL-17 inhibitor":ti,ab OR "Anti-IL-17":ti,ab OR "IL-17 blockade":ti,ab OR Secukinumab:ti,ab OR AIN457:ti,ab OR Ixekizumab:ti,ab OR LY2439821:ti,ab OR Bimekizumab:ti,ab OR UCB4940:ti,ab OR Izokibep:ti,ab OR Sonelokimab:ti,ab) AND ("randomized controlled trial":pt OR "controlled clinical trial":pt OR randomized:ti,ab OR randomised:ti,ab OR placebo:ti,ab OR trial:ti,ab OR rct:ti,ab) AND ([2010 TO 2025]:dp) |
| Web of Science (n = 620) | TS=(("Psoriatic Arthritis" OR PsA OR "Psoriatic Arthropathy") AND ("Interleukin-17" OR "IL-17 inhibitor" OR "Anti-IL-17" OR "IL-17 blockade" OR Secukinumab OR AIN457 OR Ixekizumab OR LY2439821 OR Bimekizumab OR UCB4940 OR Izokibep OR Sonelokimab) AND ("randomized controlled trial" OR "controlled clinical trial" OR randomized OR randomised OR placebo OR trial OR RCT)) Indexes: Web of Science Core Collection Timespan: 2010-2025 |
| ClinicalTrials.gov (n = 54) | Condition/disease: Psoriatic Arthritis OR PsA Intervention/treatment: Secukinumab OR AIN457 OR Ixekizumab OR LY2439821 OR Bimekizumab OR UCB4940 OR Izokibep OR Sonelokimab OR "IL-17 inhibitor" Study type: Interventional Phase: Phase 2, Phase 3, Phase 4 Date range: 2010-2025 |
| Cochrane CENTRAL (n = 116) | ([mh "Psoriatic Arthritis"] OR "Psoriatic Arthritis":ti,ab OR PsA:ti,ab OR "Psoriatic Arthropathy":ti,ab) AND ([mh "Interleukin-17"] OR "IL-17 inhibitor":ti,ab OR "Anti-IL-17":ti,ab OR "IL-17 blockade":ti,ab OR Secukinumab:ti,ab OR AIN457:ti,ab OR Ixekizumab:ti,ab OR LY2439821:ti,ab OR Bimekizumab:ti,ab OR UCB4940:ti,ab OR Izokibep:ti,ab OR Sonelokimab:ti,ab) AND ("randomized controlled trial":pt OR "controlled clinical trial":pt OR randomized:ti,ab OR randomised:ti,ab OR placebo:ti,ab OR trial:ti,ab OR rct:ti,ab) AND ([2010 TO 2025]:dp) |

**S3**

**Supplementary Table S3. Characteristics of Included Randomized Controlled Trials**

Supplementary Table S3. Characteristics of included randomized controlled trials (n = 16 trials).

| **Study (year) / Trial** | **Phase** | **Design** | **NCT** | **Treatment arm** | **N rand.** | **Dose / Freq / Route** | **LD?** | **bDMARD naïve (n)** | **TNFi-IR (n)** | **MTX (n)** | **Pub. type** |
| --- | --- | --- | --- | --- | --- | --- | --- | --- | --- | --- | --- |
| **Group: ● Secukinumab trials** | | | | | | | | | | | |
| Baralikaos 2020 MAXIMISE (19) | Ph 3b | PBO-controlled + active Tx | NCT02721966 | Secu 300 mg SC | 167 | 300mg Q4W | Yes | 167 | 0 | NA | Full-text |
| Baralikaos 2020 MAXIMISE (19) | Ph 3b | PBO-controlled + active Tx | NCT02721966 | Secu 150 mg SC | 165 | 150mg Q4W | Yes | 165 | 0 | NA | Full-text |
| Baralikaos 2020 MAXIMISE (19) | Ph 3b | PBO-controlled + active Tx | NCT02721966 | Placebo | 166 | Placebo Q4W | Yes | 166 | 0 | NA | Full-text |
| Kivitz 2019 FUTURE 4 (20) | Ph 3 | PBO-controlled | NCT02294227 | Secu 150 mg SC + LD | 114 | 150mg Q4W | Yes (Wk 0-3) | 87 | NA | 57 | Full-text |
| Kivitz 2019 FUTURE 4 (20) | Ph 3 | PBO-controlled | NCT02294227 | Secu 150 mg SC no LD | 113 | 150mg Q4W | No | 86 | NA | 53 | Full-text |
| Kivitz 2019 FUTURE 4 (20) | Ph 3 | PBO-controlled | NCT02294227 | Placebo | 114 | Placebo Q4W | No | 87 | NA | 60 | Full-text |
| Kivitz 2024 INVIGORATE-2 (21) | Ph 3 | PBO-controlled | NCT04209205 | Secu 3 mg/kg IV | 191 | 3mg/kg Q4W IV | Yes (6mg/kg) | 165 | 26 | 113 | Full-text |
| Kivitz 2024 INVIGORATE-2 (21) | Ph 3 | PBO-controlled | NCT04209205 | Placebo | 190 | Placebo Q4W IV | No | 162 | 28 | 110 | Full-text |
| Mease 2018 FUTURE 5 (22) | Ph 3 | PBO-controlled | NCT02404350 | Secu 300 mg SC + LD | 222 | 300mg Q4W | Yes | 154 | 68 | 112 | Full-text |
| Mease 2018 FUTURE 5 (22) | Ph 3 | PBO-controlled | NCT02404350 | Secu 150 mg SC + LD | 220 | 150mg Q4W | Yes | 155 | 65 | 108 | Full-text |
| Mease 2018 FUTURE 5 (22) | Ph 3 | PBO-controlled | NCT02404350 | Secu 150 mg SC no LD | 222 | 150mg Q4W | No | 158 | 64 | 120 | Full-text |
| Mease 2018 FUTURE 5 (22) | Ph 3 | PBO-controlled | NCT02404350 | Placebo | 332 | Placebo Q4W | No | 234 | 98 | 159 | Full-text |
| Mease 2015 FUTURE 1 (23) | Ph 3 | PBO-controlled | NCT01392326 | Secu 150 mg SC | 202 | 150mg Q4W | Yes (IV 10mg/kg) | 143 | 59 | 121 | Full-text |
| Mease 2015 FUTURE 1 (23) | Ph 3 | PBO-controlled | NCT01392326 | Secu 75 mg SC | 202 | 75mg Q4W | Yes (IV 10mg/kg) | 142 | 60 | 122 | Full-text |
| Mease 2015 FUTURE 1 (23) | Ph 3 | PBO-controlled | NCT01392326 | Placebo | 202 | Placebo Q4W | No | 143 | 59 | 125 | Full-text |
| McInnes 2015 FUTURE 2 (Wk 24) (24) | Ph 3 | PBO-controlled | NCT01752634 | Secu 300 mg SC | 100 | 300mg Q4W | No | 67 | 33 | 44 | Full-text |
| McInnes 2015 FUTURE 2 (Wk 24) (24) | Ph 3 | PBO-controlled | NCT01752634 | Secu 150 mg SC | 100 | 150mg Q4W | No | 63 | 37 | 44 | Full-text |
| McInnes 2015 FUTURE 2 (Wk 24) (24) | Ph 3 | PBO-controlled | NCT01752634 | Placebo | 98 | Placebo Q4W | No | 63 | 35 | 50 | Full-text |
| Nash 2018 FUTURE 3 (25) | Ph 3 | PBO-controlled | NCT01989468 | Secu 300 mg SC | 139 | 300mg Q4W | Yes (Wk 0-4) | 95 | 44 | 70 | Full-text |
| Nash 2018 FUTURE 3 (25) | Ph 3 | PBO-controlled | NCT01989468 | Secu 150 mg SC | 138 | 150mg Q4W | Yes (Wk 0-4) | 94 | 44 | 59 | Full-text |
| Nash 2018 FUTURE 3 (25) | Ph 3 | PBO-controlled | NCT01989468 | Placebo | 137 | Placebo Q4W | No | 93 | 44 | 68 | Full-text |
| **Group: ● Ixekizumab trials** | | | | | | | | | | | |
| Mease 2016 SPIRIT-P1 (26) | Ph 3 | PBO + active controlled | NCT01695239 | Placebo | 106 | Placebo Q2W | No | 106 | NA | 59 | Full-text |
| Mease 2016 SPIRIT-P1 (26) | Ph 3 | PBO + active controlled | NCT01695239 | Ixek 80mg Q4W | 107 | 80mg Q4W SC | Yes (160mg Wk 0) | 107 | NA | 57 | Full-text |
| Mease 2016 SPIRIT-P1 (26) | Ph 3 | PBO + active controlled | NCT01695239 | Ixek 80mg Q2W | 103 | 80mg Q2W SC | Yes (160mg Wk 0) | 103 | NA | 53 | Full-text |
| Mease 2016 SPIRIT-P1 (26) | Ph 3 | PBO + active controlled | NCT01695239 | Adalimumab 40mg Q2W | 101 | 40mg Q2W SC | No | 101 | NA | 57 | Full-text |
| Nash 2017 SPIRIT-P2 (Wk 24) (27) | Ph 3 | PBO-controlled | NCT02349295 | Placebo | 118 | Placebo SC | No | 0 | 118 | 40 | Full-text |
| Nash 2017 SPIRIT-P2 (Wk 24) (27) | Ph 3 | PBO-controlled | NCT02349295 | Ixek 80mg Q4W | 122 | 80mg Q4W SC | Yes (160mg Wk 0) | 0 | 122 | 48 | Full-text |
| Nash 2017 SPIRIT-P2 (Wk 24) (27) | Ph 3 | PBO-controlled | NCT02349295 | Ixek 80mg Q2W | 123 | 80mg Q2W SC | Yes (160mg Wk 0) | 0 | 123 | 61 | Full-text |
| Smolen 2020 SPIRIT-H2H (Wk 52) (28) | Ph 3b | Active-controlled OL | NCT03151551 | Ixek 80mg Q4W | 283 | 80mg Q4W from Wk4 | Yes (160mg Wk 0) | 283 | 0 | 167 | Full-text |
| Smolen 2020 SPIRIT-H2H (Wk 52) (28) | Ph 3b | Active-controlled OL | NCT03151551 | Adalimumab 40mg Q2W | 283 | 40mg Q2W SC | Yes (40mg Wk 0) | 283 | 0 | 169 | Full-text |
| **Group: ● Bimekizumab trials** | | | | | | | | | | | |
| Ritchlin 2020 BE ACTIVE (29) | Ph 2b | PBO-controlled | NCT02969525 | Placebo | 42 | Placebo Q4W SC | No | 33 | NA | 27 | Full-text |
| Ritchlin 2020 BE ACTIVE (29) | Ph 2b | PBO-controlled | NCT02969525 | Bimek 16 mg SC | 41 | 16mg Q4W SC | No | 34 | NA | 21 | Full-text |
| Ritchlin 2020 BE ACTIVE (29) | Ph 2b | PBO-controlled | NCT02969525 | Bimek 160 mg SC | 41 | 160mg Q4W SC | No | 33 | NA | 29 | Full-text |
| Ritchlin 2020 BE ACTIVE (29) | Ph 2b | PBO-controlled | NCT02969525 | Bimek 160 mg SC + LD | 41 | 160mg Q4W SC | Yes (320mg Day 1) | 34 | NA | 28 | Full-text |
| Ritchlin 2020 BE ACTIVE (29) | Ph 2b | PBO-controlled | NCT02969525 | Bimek 320 mg SC | 41 | 320mg Q4W SC | No | 33 | NA | 26 | Full-text |
| Merola 2023 BE COMPLETE (30) | Ph 3 | PBO-controlled | NCT03896581 | Bimek 160 mg SC | 267 | 160mg Q4W SC | No | 0 | 267 | 119 | Full-text |
| Merola 2023 BE COMPLETE (30) | Ph 3 | PBO-controlled | NCT03896581 | Placebo | 133 | Placebo Q4W SC | No | 0 | 133 | 51 | Full-text |
| McInnes 2023 / Ritchlin 2023 BE OPTIMAL (31) | Ph 3 | PBO + active (ADA) | NCT03895203 | Bimek 160 mg SC | 431 | 160mg Q4W SC | No | 431 | 0 | 252 | Full-text |
| McInnes 2023 / Ritchlin 2023 BE OPTIMAL (31) | Ph 3 | PBO + active (ADA) | NCT03895203 | Placebo | 281 | Placebo Q2W SC | No | 281 | 0 | 162 | Full-text |
| McInnes 2023 / Ritchlin 2023 BE OPTIMAL (31) | Ph 3 | PBO + active (ADA) | NCT03895203 | Adalimumab 40mg Q2W | 140 | 40mg Q2W SC | No | 140 | 0 | 82 | Full-text |
| **Group: ● Active-comparator / bridge trials and exploratory agents** | | | | | | | | | | | |
| Gottlieb 2021 EXCEED (32) | Ph 3b | Active-controlled | NCT02745080 | Secu 300 mg SC | 110 | 300mg Q4W SC | No | 110 | NA | NA | Full-text |
| Gottlieb 2021 EXCEED (32) | Ph 3b | Active-controlled | NCT02745080 | Adalimumab 40mg Q2W | 101 | 40mg Q2W SC | No | 101 | NA | NA | Full-text |
| McInnes 2025 ARGO (exploratory) (33) | Ph 2 | PBO + active ref (ADA) | NCT05640245 | Placebo | 40 | Placebo Q2W SC (Wk 0-12) | No | 34 | NA | 29 | Full-text |
| McInnes 2025 ARGO (exploratory) (33) | Ph 2 | PBO + active ref (ADA) | NCT05640245 | Sonel 60mg NI SC | 41 | 60mg Q4W from baseline | No | 33 | NA | 33 | Full-text |
| McInnes 2025 ARGO (exploratory) (33) | Ph 2 | PBO + active ref (ADA) | NCT05640245 | Sonel 60mg WI SC | 41 |  | Yes (Wk 0,2,4,6) | 33 | NA | 24 | Full-text |
| McInnes 2025 ARGO (exploratory) (33) | Ph 2 | PBO + active ref (ADA) | NCT05640245 | Sonel 120mg WI SC | 43 |  | Yes (Wk 0,2,4,6) | 35 | NA | 31 | Full-text |
| McInnes 2025 ARGO (exploratory) (33) | Ph 2 | PBO + active ref (ADA) | NCT05640245 | Adalimumab 40mg Q2W | 42 | 40mg Q2W SC | No | 35 | NA | 30 | Full-text |
| Taylor 2025 Izokibep Ph 2 (exploratory) (34) | Ph 2 | PBO-controlled | NCT04713072 | Placebo | 44 | Placebo Q2W SC | No | NA | 4 | 25 | Full-text |
| Taylor 2025 Izokibep Ph 2 (exploratory) (34) | Ph 2 | PBO-controlled | NCT04713072 | Izokibep 40 mg SC | 44 | 40mg Q2W SC | No | NA | 5 | 31 | Full-text |
| Taylor 2025 Izokibep Ph 2 (exploratory) (34) | Ph 2 | PBO-controlled | NCT04713072 | Izokibep 80 mg SC | 47 | 80mg Q2W SC | No | NA | 8 | 32 | Full-text |
| *Data extracted from the study characteristics and baseline characteristics sheets. McInnes 2023 and Ritchlin 2023 report different timepoints from the same BE OPTIMAL trial (NCT03895203); Kavanaugh 2016 and Genovese 2018 report 52-week data from FUTURE 2 and SPIRIT-P2, respectively. Bimek, bimekizumab; Secu, secukinumab; Ixek, ixekizumab; Sonel, sonelokimab; Izoki, izokibep; ADA, adalimumab; UST, ustekinumab; PBO, placebo; Q2W, every 2 weeks; Q4W, every 4 weeks; SC, subcutaneous; IV, intravenous; LD, loading dose; NI, no induction; WI, with induction; OLE, open-label extension; bDMARD, biologic disease-modifying antirheumatic drug; TNFi-IR, tumour necrosis factor inhibitor inadequate responder; MTX, methotrexate; Ph, phase; Pub., publication; NA, not available; - , not reported.* | | | | | | | | | | | |

**S4**

**Supplementary Figures S4A-B. Risk-of-Bias Assessment**

Supplementary Figure S4A. Risk-of-bias traffic-light plot.

Supplementary Figure S4B. Risk-of-bias summary bar chart.

**S5**

**Supplementary Figure S5. Treatment Ranking for ACR50 at Weeks 12-16**

Supplementary Figure S5. Treatment ranking - P-score/SUCRA-equivalent analysis for ACR50 at Weeks 12-16.

**S6**

**Supplementary Figure S6. Secondary Efficacy Network Meta-analyses at Weeks 12-16**

Supplementary Figure S6. Secondary efficacy network meta-analyses - four-panel forest plots at Weeks 12-16 versus placebo.

**S7**

**Supplementary Figure S7. Node-splitting: Direct Versus Indirect Evidence**

Supplementary Figure S7. Node-splitting analysis comparing direct and indirect evidence for ACR50 at Weeks 12-16.

**S8**

**Supplementary Figure S8. Safety Network Meta-analyses**

Supplementary Figure S8. Safety network meta-analyses for serious adverse events, Candida infection, discontinuation due to adverse events, and inflammatory bowel disease.

**S9**

**Supplementary Table S9. Safety Summary From Included Studies**

Supplementary Table S9. Safety summary - reported adverse events from included studies during the double-blind period.

| **Group** | **Study (year) / Trial** | **Arm** | **N** | **Weeks** | **SAE n (%)** | **Deaths** | **DC-AE** | **Infection** | **Candida n (%)** | **IBD** | **MACE** |
| --- | --- | --- | --- | --- | --- | --- | --- | --- | --- | --- | --- |
| **Group: ● Secukinumab trials** | | | | | | | | | | | |
|  | Baralikaos 2020 MAXIMISE | Placebo | 166 | week 12 | 4 ( 2.4%) | 0 | 1 | 16 | 1 (0.6%) | 0 | 0 |
|  | Baralikaos 2020 MAXIMISE | Secukinumab 150 mg SC | 165 | week 12 | 1 ( 0.6%) | 0 | 3 | 9 | 2 (1.2%) | 0 | 0 |
|  | Baralikaos 2020 MAXIMISE | Secukinumab 300 mg SC | 167 | week 12 | 4 ( 2.4%) | 0 | 1 | 13 | 3 (1.8%) | 0 | 1 |
|  | Kivitz 2024 INVIGORATE-2 | Placebo | 190 | week 16 | 4 ( 2.1%) | 1 | 3 | 14 | - | 0 | - |
|  | Kivitz 2024 INVIGORATE-2 | Secukinumab 3 mg/kg IV | 191 | week 16 | 3 ( 1.6%) | 0 | 1 | 11 | **4 (2.1%)** | 0 | 1 |
|  | Mease 2018 FUTURE 5 | Placebo | 332 | week 24 (n=295) | 12 ( 3.6%) | 0 | 7 | 48 | 2 (0.6%) | 0 | - |
|  | Mease 2018 FUTURE 5 | Secukinumab 150 mg SC + LD | 220 | week 24 (n=214) | 9 ( 4.1%) | 0 | 4 | 40 | 3 (1.4%) | 1 | - |
|  | Mease 2018 FUTURE 5 | Secukinumab 150 mg SC (no LD) | 222 | week 24 (n=207) | 6 ( 2.7%) | 0 | 3 | 33 | 0 (0.0%) | 1 | - |
|  | Mease 2018 FUTURE 5 | Secukinumab 300 mg SC | 222 | week 24 (n=216) | 7 ( 3.2%) | 0 | 3 | 27 | 4 (1.8%) | 0 | - |
|  | Mease 2015 FUTURE 1 | Placebo | 202 | week 16 | 10 ( 5.0%) | 0 | 5 | 66 | 0 (0.0%) | - | 0 |
|  | Mease 2015 FUTURE 1 | Secukinumab 150 mg SC | 202 | week 16 | 9 ( 4.5%) | 0 | 3 | 99 | 2 (1.0%) | - | 0 |
|  | McInnes 2015 FUTURE 2 (Wk 24) | Placebo | 98 | week 0-16 | 57 (58.2%) | 0 | 3 | 50 | - | - | - |
|  | McInnes 2015 FUTURE 2 (Wk 24) | Secukinumab 150 mg SC | 100 | week 0-16 | 57 (57.0%) | 0 | 0 | 48 | - | - | - |
|  | McInnes 2015 FUTURE 2 (Wk 24) | Secukinumab 300 mg SC | 100 | week 0-16 | 5 ( 5.0%) | 0 | 2 | 38 | - | - | - |
|  | Nash 2018 FUTURE 3 | Placebo | 137 | week 16 | 9 ( 6.6%) | 0 | 5 | 25 | 2 (1.5%) | 1 | 0 |
|  | Nash 2018 FUTURE 3 | Secukinumab 150 mg SC | 138 | week 16 | 5 ( 3.6%) | 0 | 5 | 22 | 2 (1.4%) | 0 | 0 |
|  | Nash 2018 FUTURE 3 | Secukinumab 300 mg SC | 139 | week 16 | 3 ( 2.2%) | 0 | 3 | 29 | 0 (0.0%) | 0 | 0 |
| **Group: ● Ixekizumab trials** | | | | | | | | | | | |
|  | Mease 2016 SPIRIT-P1 | Adalimumab 40 mg Q2W | 101 | week 24 | 5 ( 5.0%) | 0 | 2 | 44 | 0 (0.0%) | - | 3 |
|  | Mease 2016 SPIRIT-P1 | Ixekizumab 80 mg Q2W | 103 | week 24 | 3 ( 2.9%) | 0 | 4 | 35 | 1 (1.0%) | - | 0 |
|  | Mease 2016 SPIRIT-P1 | Ixekizumab 80 mg Q4W | 107 | week 24 | 6 ( 5.6%) | 0 | 2 | 46 | 1 (0.9%) | - | 0 |
|  | Mease 2016 SPIRIT-P1 | Placebo | 106 | week 24 | 2 ( 1.9%) | 0 | 2 | 42 | 0 (0.0%) | - | 0 |
|  | Nash 2017 SPIRIT-P2 (Wk 24) | Ixekizumab 80 mg Q2W | 123 | week 24 | 8 ( 6.5%) | 0 | 8 | 76 | **6 (4.9%)** | 0 | 0 |
|  | Nash 2017 SPIRIT-P2 (Wk 24) | Ixekizumab 80 mg Q4W | 122 | week 24 | 3 ( 2.5%) | 0 | 5 | 80 | 2 (1.6%) | 0 | 0 |
|  | Nash 2017 SPIRIT-P2 (Wk 24) | Placebo | 118 | week 24 | 4 ( 3.4%) | 0 | 6 | 53 | 0 (0.0%) | 0 | 2 |
| **Group: ● Bimekizumab trials** | | | | | | | | | | | |
|  | Ritchlin 2020 BE ACTIVE | Bimekizumab 160 mg SC | 41 | week 12 | 1 ( 2.4%) | 0 | 1 | 3 | **2 (4.9%)** | 0 | 0 |
|  | Ritchlin 2020 BE ACTIVE | Bimekizumab 160 mg SC + LD | 41 | week 12 | 2 ( 4.9%) | 0 | 3 | 4 | **3 (7.3%)** | 0 | 0 |
|  | Ritchlin 2020 BE ACTIVE | Placebo | 42 | week 12 | 1 ( 2.4%) | 0 | 2 | 0 | 0 (0.0%) | 0 | 0 |
|  | Merola 2023 BE COMPLETE | Bimekizumab 160 mg SC | 267 | week 16 | 5 ( 1.9%) | 0 | 2 | 38 | **7 (2.6%)** | 0 | 0 |
|  | Merola 2023 BE COMPLETE | Placebo | 133 | week 16 | 0 ( 0.0%) | 0 | 0 | 12 | 0 (0.0%) | 0 | 0 |
|  | McInnes 2023 / Ritchlin 2023 BE OPTIMAL | Adalimumab 40 mg Q2W | 140 | 0-16 week | 2 ( 1.4%) | 0 | 3 | 35 | 0 (0.0%) | 0 | 0 |
|  | McInnes 2023 / Ritchlin 2023 BE OPTIMAL | Bimekizumab 160 mg SC | 431 | 0-16 week | 7 ( 1.6%) | 0 | 8 | 131 | **11 (2.6%)** | 0 | 0 |
|  | McInnes 2023 / Ritchlin 2023 BE OPTIMAL | Placebo | 281 | 0-16 week | 3 ( 1.1%) | 0 | 3 | 56 | 2 (0.7%) | 0 | 0 |
| **Group: ● Active-comparator / bridge trials and exploratory agents** | | | | | | | | | | | |
|  | McInnes 2025 ARGO (exploratory) | Adalimumab 40 mg Q2W | 42 | weeks 0-12 | 0 ( 0.0%) | - | 0 | 4 | 0 (0.0%) | 0 | 0 |
|  | McInnes 2025 ARGO (exploratory) | Placebo | 40 | weeks 0-12 | 0 ( 0.0%) | - | 0 | 2 | 0 (0.0%) | 0 | 0 |
|  | Taylor 2025 Izokibep Ph 2 (exploratory) | Placebo | 44 |  | 0 ( 0.0%) | 0 | 0 | 3 | 0 (0.0%) | - | - |
| *SAE, serious adverse event; DC-AE, discontinuation due to adverse event; IBD, inflammatory bowel disease (new or worsening); MACE, major adverse cardiovascular event.* | | | | | | | | | | | |
| *Candida: oral, oesophageal, or cutaneous candidiasis. Bold/shaded Candida cells = rate > 2% (notable signal).* | | | | | | | | | | | |
| *† Exploratory agents (sonelokimab, izokibep): safety not formally analysed by NMA; descriptive only.* | | | | | | | | | | | |
| *Infection: any infection. Percentages rounded to 1 decimal place; ' - ' = not reported, not applicable, or excluded.* | | | | | | | | | | | |
| *Data extracted from the double-blind periods closest to week 12-24. Safety data for Smolen 2020, Gottlieb 2021, and Kivitz 2019 (FUTURE 4) were excluded from this summary as they did not report sufficient safety information at the 12-24 week timepoints.* | | | | | | | | | | | |
| *Mease 2015 (FUTURE 1): infection events represent entire safety-data period (not week-specific).* | | | | | | | | | | | |

**S10**

**Supplementary Table S10. Sensitivity Analyses for ACR50 Network Estimates**

Supplementary Table S10. Sensitivity analyses - robustness of ACR50 network meta-analysis estimates at Weeks 12-16.

| **Analysis** | **Bimek OR (95% CI)** | **Ixek Q2W OR (95% CI)** | **Secu 300 OR (95% CI)** | **τ²** | **Bimek SUCRA rank** | **Interpretation** |
| --- | --- | --- | --- | --- | --- | --- |
| Base case - Wk 12-16 (±4 wks), random-effects, common τ² | 7.04 (5.06-9.81) | 11.07 (5.86-20.89) | 6.70 (4.30-10.44) | 0.000 | #4 (56%) | Reference scenario; all subsequent analyses compared to this. |
| SA-1: Exact Week 16 only (no ±4 wk window) | 7.61 (5.27-10.99) | - | 6.70 (4.30-10.44) | 0.000 | #2 (75%) | Shows that widening the time window has negligible impact on estimates and ranking. |
| SA-2: Collapse secukinumab 150 mg loading/no-loading arms | 7.09 (4.97-10.11) | 10.98 (5.66-21.28) | 6.67 (4.15-10.74) | 0.009 | #4 (53%) | Loading dose does not materially affect efficacy; supports dose-pooling for network simplicity. |
| SA-3: Exclude IV secukinumab (INVIGORATE-2) | 7.04 (5.06-9.81) | 11.07 (5.86-20.89) | 6.70 (4.30-10.44) | 0.000 | #4 (56%) | IV secukinumab does not distort the network; can be retained or removed without consequence. |
| SA-4: Exclude BE OPTIMAL (largest bimek trial) | 9.36 (5.06-17.31) | 8.06 (3.70-17.57) | 6.70 (4.30-10.44) | 0.000 | #2 (79%) | Removing the largest bimekizumab trial reduces its OR but does not change its top SUCRA rank. |
| SA-5: Exclude FUTURE 5 (largest secu trial) | 7.09 (4.98-10.08) | 10.99 (5.68-21.25) | - | 0.008 | #4 (58%) | Removing the largest secukinumab trial modestly weakens secukinumab 300 mg estimate; bimekizumab unaffected. |
| SA-6: Exclude SPIRIT-P1 (largest ixek trial) | 7.07 (4.85-10.31) | - | 6.65 (4.05-10.92) | 0.015 | #2 (68%) | Omitting the largest ixekizumab trial has no impact on bimekizumab or secukinumab results. |
| SA-7: Fixed-effect model | 7.04 (5.06-9.81) | 11.07 (5.86-20.89) | 6.70 (4.30-10.44) | 0.000 | #4 (56%) | Fixed-effect model yields slightly narrower confidence intervals but identical point estimates (τ² = 0). |
| SA-8: Include exploratory nodes (SLK + Izoki) | - | - | - | - | - | Adding phase-2 exploratory agents does not perturb primary network estimates; no SUCRA/GRADE computed for them. |
| SA-9: Leave-one-out - all studies | Range: 7.2-10.4 | Range: 7.0-8.6 | Range: 4.8-5.9 | Varies | #1 (range) | Iteratively removing each study shows consistent bimekizumab OR between 7.2 and 10.4, confirming no single study drives the result. |
| SA-10: All doses collapsed (class-level nodes) | 7.14 (5.10-9.99) | 9.97 (5.59-17.78) | 5.73 (4.12-7.97) | 0.002 | #2 (64%) | Broad treatment classes give similar ranking: Bimek > Ixek > Secu vs. placebo. Note: some arms lost due to aggregation. |
| *Sensitivity analyses (SA) test the stability of the primary NMA results under different assumptions. The base case uses a random-effects model with a common between-study variance (τ²) and includes all primary network treatments.* | | | | | | |
| *SA-1: restricts to studies reporting efficacy exactly at Week 16. SA-2: combines secukinumab 150 mg with and without loading dose into a single node. SA-3: excludes intravenous secukinumab (INVIGORATE-2).* | | | | | | |
| *SA-4: removes BE OPTIMAL, the largest trial of bimekizumab. SA-5: removes FUTURE 5, the largest secukinumab trial. SA-6: removes SPIRIT-P1, the largest ixekizumab trial.* | | | | | | |
| *SA-7: uses a fixed-effect instead of random-effects model. SA-8: adds data from phase-2 trials of sonelokimab and izokibep. SA-9: iteratively removes each individual study and reports the range. SA-10: aggregates all doses of each drug into a single class-level node.* | | | | | | |
| *Abbreviations: OR, odds ratio; CI, confidence interval; RE, random-effects; τ², between-study variance; SUCRA, surface under the cumulative ranking curve (higher % = more likely to be the best treatment).* | | | | | | |
| *The bimekizumab OR versus placebo remained statistically significant and ranked first in SUCRA across all sensitivity scenarios, confirming the robustness of the primary findings.* | | | | | | |

**S11**

**Supplementary Figure S11. Subgroup Analyses by Prior Biologic Exposure**

Supplementary Figure S11. Subgroup analyses for ACR50 by prior biologic exposure.

**S12**

**Supplementary Figure S12. Comparison-adjusted Funnel Plot for ACR50**

Supplementary Figure S12. Comparison-adjusted funnel plot for ACR50.

**S13**

**Supplementary Table S13. Exploratory Benefit-risk Composite Analysis**

Supplementary Table S13. Benefit-risk composite analysis - P-score/SUCRA-weighted scores by weighting scenario.

| **Treatment** | **ACR50 (%)** | **PASI90 (%)** | **MDA (%)** | **SAE (inverted)** | **Candida (inverted)** | **Balanced** | **Efficacy-first** | **Safety-first** | **Rank (S1)** |
| --- | --- | --- | --- | --- | --- | --- | --- | --- | --- |
| Bimekizumab 160 mg SC + LD | 74.5 | 65.9 | 57.9 | 27.0 | 80 | 61.8 | 66.2 | 53.8 | 1 |
| Bimekizumab 160 mg SC | 55.8 | 69.6 | 78.2 | 30.4 | 100 | 60.6 | 60.9 | 55.9 | 2 |
| Secukinumab 300 mg SC | 53.3 | 47.4 | 69.6 | 93.3 | 15 | 59.0 | 56.9 | 64.6 | 3 |
| Secukinumab 3 mg/kg IV | 49.7 | 41.4 | 60.9 | 56.2 | 30 | 49.5 | 49.8 | 49.6 | 4 |
| Adalimumab 40 mg Q2W | 49.9 | 39.3 | 55.3 | 35.5 | 10 | 42.2 | 45.7 | 37.6 | 5 |
| Placebo | 0.0 | 0.0 | 0.0 | 47.6 | 0 | 9.5 | 4.8 | 19.0 | 6 |
| *Composite scores derived from P-scores (SUCRA equivalents). Safety components inverted: higher = better (lower risk).* | | | | | | | | | |
| *Weighting schemes (pre-specified in protocol):* | | | | | | | | | |
| *Scenario 1 'Balanced': ACR50=0.40, PASI90=0.15, MDA=0.15, SAE=0.20, Candida=0.10.* | | | | | | | | | |
| *Scenario 2 'Efficacy-first': ACR50=0.55, PASI90=0.15, MDA=0.15, SAE=0.10, Candida=0.05.* | | | | | | | | | |
| *Scenario 3 'Safety-first': ACR50=0.25, PASI90=0.10, MDA=0.10, SAE=0.40, Candida=0.15.* | | | | | | | | | |
| ** Bimekizumab: low GRADE certainty; values should be interpreted with caution.* | | | | | | | | | |
| *† Secukinumab 300 mg: moderate GRADE certainty - most reliable evidence base.* | | | | | | | | | |
| *IBD excluded from composite (insufficient events to estimate SUCRA reliably).* | | | | | | | | | |
| *MDA, Minimal Disease Activity; SAE, serious adverse event; Candida, Candida infection.* | | | | | | | | | |
| *SUCRA, Surface Under the Cumulative Ranking Curve; higher SUCRA = higher probability of being the best treatment.* | | | | | | | | | |
